# Pentanucleotide guanine-rich WGGGW repeats, including CANVAS AGGGA repeats, form a variety of noncanonical structures

**DOI:** 10.1093/nar/gkag051

**Published:** 2026-01-30

**Authors:** Jiawei Wang, Dehui Qiu, Jun Zhou, Jean-Louis Mergny, Patrizia Alberti

**Affiliations:** Laboratoire d’Optique et Biosciences, Ecole Polytechnique, CNRS, INSERM, Institut Polytechnique de Paris, 91120 Palaiseau cedex, France; State Key Laboratory of Analytical Chemistry for Life Science, School of Chemistry and Chemical Engineering, Nanjing University, Nanjing 210023, P.R. China; State Key Laboratory of Analytical Chemistry for Life Science, School of Chemistry and Chemical Engineering, Nanjing University, Nanjing 210023, P.R. China; Laboratoire d’Optique et Biosciences, Ecole Polytechnique, CNRS, INSERM, Institut Polytechnique de Paris, 91120 Palaiseau cedex, France; Laboratoire Structure et Instabilité des Génomes, Muséum national d’Histoire naturelle, CNRS, INSERM, Sorbonne Université, 75005 Paris, France

## Abstract

Short tandem repeats (STRs) are an important component of the human genome as they contribute to genetic diversity and can influence gene expression and disease susceptibility. STRs are important in the context of CANVAS (Cerebellar Ataxia, Neuropathy, Vestibular Areflexia Syndrome) genetic disease as expansions of AGGGA repeats within the RFC1 gene are associated with the development of this neurodegenerative disorder. Interestingly, the RFC1 expanded motifs are pentanucleotides that differ from the nonpathogenic AGAAA pentanucleotide motif present in reference genomes. The molecular mechanisms underlying the pathogenicity of the mutated pentanucleotide expansion in CANVAS are still unknown. Several groups have shown that DNA and RNA containing AGGGA repeats fold into G-quadruplexes (G4s) under physiological K⁺ conditions. In this study, we reveal a more complex than expected behavior, in which DNA WGGGW motifs (where W is A or T) may adopt different G4 and non-G4 structures depending on sequence, repeat number and ionic conditions. These findings are relevant as they may help explain the genomic instability and pathogenicity specifically associated with AGGGA repeats among the WGGGW motifs.

## Graphical Abstract

**Graphical Abstract:**
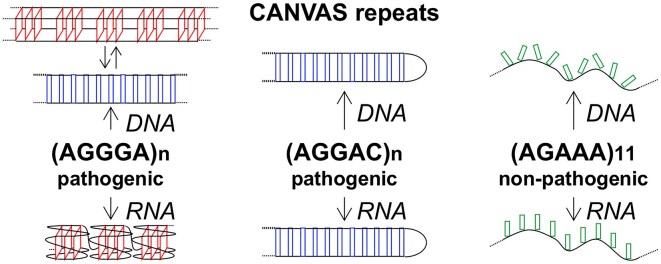

## Introduction

Nucleic acids with peculiar sequences can fold, under certain environmental conditions, into noncanonical structures, including left- or right-handed double-stranded structures with noncanonical base pairing, three-stranded structures (e.g. pyrimidine and purine triplexes), and four-stranded structures (G-quadruplexes and i-motifs). In genomes, noncanonical DNA structures may act as functional elements (e.g. G-quadruplexes in promoters [1] or in replication origins [2]) or constitute a source of genomic instability due to their mutagenic potential. Genomic instability, in turn, can lead to genomic diversity (and thus be a driver of genomic evolution) as well as to disease [3].

A class of DNA sequences that tend to fold into noncanonical structures are short tandem repeats (STRs), also known as microsatellites. These sequences consist of tandem repeats of motifs of 1–6 nucleotides in length. The propensity of STRs to adopt secondary structures is sequence-dependent and has been correlated with their potential to stall polymerase, leading to different outcomes in STR length dynamics [4]. The features of STRs vary across organisms [5]; in humans, STRs constitute ∼3% of the human genome [6]. STR expansions in several loci in the human genome underlie a number of neurodegenerative diseases [7]. Disease-associated STR expansions can be located in exons, in introns or in the 3′ and 5′ untranslated regions of a messenger RNA. The relationship between STR repeat length and pathological thresholds is complex. The expansions found in coding regions range from a few dozen to a few hundred of repeats, while those found in noncoding regions range from tens to thousands of repeats [8]. A variety of molecular mechanisms may explain the repeat-mediated genomic instability and cellular toxicity [9]. The first pathogenic STR expansions, identified about thirty years ago, were trinucleotide repeats. Since then, advances in sequencing technologies have significantly accelerated the discovery and analysis of new STR expansions, leading to the identification of tetra, penta, hexa and even dodecanucleotide repeat expansions [8].

A new class of pathogenic STR expansions has emerged in the past 15 years; its peculiar characteristic is that the expanded motifs are pentanucleotides that differ from the nonpathogenic pentanucleotide motifs present in reference genomes. This type of pentanucleotide expansions underlies several disorders: SCA37 (spinocerebellar ataxia) and six FAME (familial adult myoclonic epilepsy) disorders, where expanded ATTTC repeats are inserted into or follow nonpathogenic ATTTT repeats, and SCA31, where nonpathogenic TAAAA repeats are replaced by expanded TGGAA repeats [10]. With the exception of FAME7, these pentanucleotide expansions are located in the poly(A) regions of Alu elements [10].

An additional disorder associated with this type of pentanucleotide expansion is CANVAS (cerebellar ataxia, neuropathy and vestibular areflexia syndrome), which is the only disease known to date where the repeat motif has switched and expanded from a nonpathogenic A-rich motif to a pathogenic G-rich motif. Biallelic AGGGA repeat expansions, ranging in size from 400 to 2000 repeats, have been recently identified as the genetic anomaly that characterizes the late-onset neurogenerative disease CANVAS [11, 12]. These repeats are located in the poly(A) region of an AluSx3 element in intron 2 of the RFC1 gene, which encodes the large subunit of replication factor C (a DNA polymerase accessory protein). In healthy individuals, the more frequent configuration of this locus consists of eleven AAAAG repeats (75%); less frequent configurations consist of 15-200 AAAAG repeats (13%) or 40-1000 AAAGG repeats (8%), while <1% of healthy individuals are heterozygous for AGGGA expansions (400–2000 repeats) [11]. A second pathogenic configuration consisting of biallelic ACAGG repeat expansions has been identified in Asian CANVAS patients [13, 14]. Haplotype analysis of CANVAS cohorts has revealed that the AGGGA variant emerged in Europe >25,000 years ago, and that the ACAGG variant exhibited enrichment among non-European populations during the same period [12, 13]. Other nonpathogenic and pathogenic configurations have been identified, further highlighting the polymorphism of this locus in both healthy and CANVAS populations [15–17]. More complex genotypes associated with CANVAS (such as compound heterozygous for AGGGA expansions and truncating mutations in the RFC1 gene) have also been discovered [18, 19]. The molecular mechanisms underlying the pathogenicity of mutated pentanucleotide expansions in CANVAS are still unknown. They do not appear to be related to known mechanisms that underlie the pathogenicity of other repeat expansions [11, 20]. A recent study suggests a repeat-dependent but RFC1 protein-independent mechanism, and opens interesting directions for future research, proposing that the Alu element where these repeats are inserted may have functions that extend beyond the RFC1 gene [20]. Since the identification of biallelic expansion of AGGGA repeats in CANVAS patients [11, 12], complex biallelic pentanucleotide repeat expansions in the RFC1 gene have been detected in other neurogenerative diseases, including Parkinson disease [21–26].

Due to the propensity of sequences containing runs of consecutive guanines to fold into G-quadruplexes (G4s), several studies have promptly investigated the structures formed by DNA and RNA pentanucleotide repeats involved in CANVAS (the pathogenic AGGGA and ACAGG repeats and the normal AAAAG repeats). *In vitro* studies showed that DNA and RNA containing AGGGA repeats fold into G4s under physiological K⁺ conditions, while the A- and T-rich AAAAG/CTTTT repeats lack stable higher-order folds. Nonetheless, most of the studies focused on four repeats and yielded nonunivocal and sometimes conflicting conclusions regarding the molecularity of the proposed G4 structures formed by AGGGA repeats in the presence of K^+^, thus preventing the drawing of a clear and comprehensive picture of the structures formed by DNA and RNA AGGGA. Abdi *et al.* reported that four DNA and RNA AGGGA repeats folded into intermolecular parallel G4s [27], while Kudo *et al.* proposed the formation of an intramolecular hybrid-type G4 by four dAGGGA repeats and of intramolecular parallel G4s by four and eleven rAGGGA repeats [28]. Wang *et al.*, beside resolving the structure formed in solution by two dAGGGA repeats (a bimolecular parallel G4), reported that four dAGGGA repeats tended to fold into an intermolecular G4, while eight dAGGGA repeats folded into an intramolecular parallel G4, as well as four rAGGGA repeats [29]. Unlike AGGGA repeats, four repeats of the normal motif dAAAAG did not appear to be structured in the presence of K^+^ and at neutral pH [27], while four rAAAAG repeats displayed a circular dichroism (CD) spectrum reminiscent of single-stranded poly(A) helical structures [28]. The structure of the pathogenic ACAGG repeats has also been investigated: unlike AGGGA repeats, four DNA or RNA ACAGG repeats did not show a characteristic CD signature, while eleven rACAGG repeats folded into hairpins [28]. Beside G4s, DNA AAGGG/CCCTT repeats have been shown to fold into triplexes: a pyrimidine triplex d(CCCTT)_4_•d(AAGGG)_4_-*d(CCCTT)*_4_ in NaCl solutions at acidic pH [27], and a purine triplex d(CCCTT)_4_•d(AAGGG)_4_-*d(AAGGG)*_4_ in the presence of Li^+^ and Mg^2+^ at neutral pH [28].

Longer AGGGA repeats have been studied for their effects on biological processes. Unlike AAAAG repeats, ten AGGGA repeats have been shown to stall polymerases *in vitro*; the observed stalling pattern was found to be consistent with the formation of a purine triplex or a G4 structure, depending on the ion present in solution (Mg^2+^ or K^+^, respectively) [30]. In both yeast and human cell contexts, a run of sixty AGGGA repeats has been shown to induce replication fork stalling only when located on the lagging strand template [30], similarly to what was first observed in a yeast system for Friedreich’s ataxia-associated GAA repeats expansions [31].

Taken together, these studies highlight the potential of the pathological AGGGA repeats to fold into G4s and triplexes at the DNA level (depending on ionic and pH conditions) and suggest the implication of these DNA structures in impairing replication, a mechanism that may contribute to AGGGA expansions.

Despite this relative rich literature on CANVAS AGGGA repeats, we sought to further investigate the folding behavior of these repeats. We hypothesized that this purine-only repeated motif might enable structural heterogeneity—the ability to adopt more than one stable conformation. A DNA strand composed solely of purines may engage in noncanonical duplex arrangements through purine–purine pairing. These noncanonical duplexes can be stable under certain conditions, with protonation or specific ions sometimes contributing to their stabilization; they constitute one of the possible folding modes for homopurine strands, together with G4 folds and single-stranded forms. In order to achieve a more comprehensive understanding of the structures formed by CANVAS-associated repeats, we designed series of oligonucleotides composed of an increasing number (from 4 to 16) of AGGGA, AGGAC, and AGAAA repeats. The CANVAS AGGGA motif is one of the four WGGGW possible motifs (where W is A or T). Nevertheless, while dAGGGA, dAGGGT, dTGGGA, and dTGGGT motifs may all be found in the human genome, to the best of our knowledge only dAGGGA expansions have been involved in pathogenic processes. To investigate whether the folding behaviour of dAGGGA repeats diverged from that of the other dWGGGW repeats, we extended our analyses to include dWGGGW repeats. We used a combination of biophysical and biochemical methods to probe the conformations of these repeats under physiological potassium conditions as well as nonphysiological lithium-only conditions. Our study reveals the structural diversity exhibited by dWGGGW repeats, depending on the number of repeats, the identity of the W nucleotides (A or T) and ionic conditions, and provides a better understanding of the conformational landscape of AGGGA repeats. We show how a change in a single nucleotide within a repeated motif influence the outcome of possible structures. Notably, we found that the behaviour of dAGGGA repeats was more complex than the other dWGGGW repeats, and also more complex than previously reported, as it could not be explained by the formation of only G4s. Our demonstration that dAGGGA repeats can adopt at least two distinct folds (G4s and duplexes) highlights a case of structural polymorphism with potential implications for genomic stability and might explain why the AGGGA motif, uniquely among the WGGGW family, is associated with pathogenicity.

## Materials and methods

### Oligonucleotides

Oligonucleotides were purchased from Eurogentec (Belgium) in the form of dried powder, dissolved in bidistilled water at a concentration of 200 μM and stored at −20°C. Concentrations were determined by absorbance at 260 nm using the molar extinction coefficients provided by the manufacturer. DNA oligonucleotides used for UV-spectroscopy and gel electrophoresis were RP-Cartridge Gold^TM^ purified (if ≤40 nt in length) or polyacrylamide gel electrophoresis (PAGE) purified (if >40 nt in length); the DNA oligonucleotide used for nuclear magnetic resonance (NMR) was purified by high performance liquid chromatography reverse phase (HPLC-RP); RNA oligonucleotides were HPLC-RP purified (if ≤40 nt in length) or PAGE purified (if >40 nt in length). The sequences of all oligonucleotides used in this study are reported in Supplementary Table S1.

### Iso-FRET competition assay

Isothermal fluorescence resonance energy transfer (Iso-FRET) competition assay was carried out in 96-well plates, in a final volume of 25 μl, on an Infinite M1000 PRO microplate reader (Tecan) according to the protocol previously described [32]. Five microlitres of competitor oligonucleotide at a concentration of 100/*n* μM (where *n* is the number of repeats for pentanucleotide repeats and *n* = 4 for the control oligonucleotides VEGF, ss23, and ds26, which are a G4, a single-stranded, and a double-stranded control, respectively) were mixed with 5 μl of 1 μM 37Q (a G4-forming RNA oligonucleotide conjugated to a BQH1 fluorescence quencher). Ten microlitres of 2.5 μM PhenDC3 were then added and left to stand for 5 min. Finally, 5 μl of 100 nM F22 (an RNA oligonucleotide, complementary to 37Q and conjugated to a FAM fluorophore) were added. The final concentrations were the following: 200 nM 37Q, 20/*n* μM of competitor oligonucleotide, 1 μM PhenDC3, 20 nM F22, in 10 mM cacodylic acid at pH 7.2 (pH adjusted with LiOH), 20 mM KCl, 80 mM LiCl, 0.4% (v/v) dimethyl sulfoxide (DMSO). The reference samples for the highest and the lowest values of fluorescence intensity contained only 20 nM F22 alone or 20 nM F22 together with 200 nM 37Q, respectively. Plates were kept at room temperature (around 25°C) for 2 h before measurements. Measurements were carried out at 20°C. FAM was excited at 492 nm and the emission wavelength was set to 517 nm, excitation and emission bandwidths were set to 5 nm and the integration time to 20 μs. Each competitor oligonucleotide was tested in triplicate.

### NMM fluorescence assay

N-methyl mesoporphyrin IX (NMM) fluorescence assays were carried out in a 96-well plate, in a final volume of 25 μl, on an Infinite M1000 PRO microplate reader (Tecan). NMM (Sigma; final concentration 5 μM) was added to oligonucleotide solutions (final concentration 10/*n* μM, where *n* is defined as above) in a cacodylic acid buffer (final concentration 10 mM) at pH 7.2 (pH adjusted with LiOH), containing KCl and/or LiCl at the final concentrations reported in the figure legends. After adding NMM, the samples were incubated at room temperature for 1 h. Measurements were carried out at 20°C. NMM was excited at 380 nm and the emission wavelength was set to 610 nm; excitation and emission bandwidths were 5 nm and the integration time was 20 μs. Each competitor oligonucleotide was tested in triplicate.

### UV-absorption and circular dichroism spectroscopy

Oligonucleotides were diluted in a 10 mM cacodylic acid buffer at pH 7.2 (pH adjusted with LiOH), supplemented with either 100 mM KCl or LiCl (unless otherwise specified in the figure legends), at a strand concentration of 24/*n* μM where *n* is the number of pentanucleotide repeats composing the studied oligonucleotides, as indicated in the figure legends. Melting curves were obtained by recording the absorbance at 335, 295, 273, 260, and 245 nm as a function of temperature: the temperature was first decreased from 95°C to 5°C at a rate of 0.2°C min^−1^, then increased from 5°C to 95°C at a rate of 0.2°C min^−1^, and finally decreased from 95°C to 5°C at a rate of 0.4°C min^−1^. Melting curves at 295, 273, 260, and 245 nm were corrected by subtracting the absorbance at 335 nm as a function of temperature. To avoid assumptions about the transition process (two-state or more complex) and about the temperature dependence of the absorbance of the structured and unstructured forms (i.e. the selection of high- and low-temperature baselines), the melting temperatures (*T*m) were estimated as the temperatures corresponding to the extrema (maximum or minimum, depending on the wavelength) of the first derivative of absorbance with respect to temperature [33]. During the acquisition of melting curves, absorbance spectra were recorded at high (95°C) and low (5°C) temperatures; thermal difference spectra (TDS) were obtained by subtracting the absorbance spectrum at low temperature (5°C) from the absorbance spectrum at high temperature (95°C). A TDS provides insights into the conformations adopted by an oligonucleotide [34]. Once the samples had reached 5°C at the end of the acquisition of melting curves, CD spectra were recorded first at 5°C, then at 85°C. Each CD spectrum was obtained by averaging three scans at a speed of 500 nm min^−1^ and was corrected by subtracting the spectrum of a water filled quartz cell. Measurements were carried out in quartz cuvettes with an optical path of 1 cm (Hellma).

### Polyacrylamide gel electrophoresis

Oligonucleotides were diluted in a 10 mM cacodylic acid buffer at pH 7.2 (pH adjusted with LiOH), containing 100 mM KCl or LiCl at strand concentrations indicated in figure legends, heated at 90°C for a few minutes and slowly cooled to 4°C. After addition of sucrose (10%), oligonucleotide solutions were loaded on polyacrylamide gels, prepared in a 1× Tris–borate–ethylenediaminetetraacetic acid (TBE) buffer supplemented with KCl or LiCl 20 mM. For UV-shadowing detection, oligonucleotides were loaded on 12% polyacrylamide gels (acrylamide:bisacrylamyde 19:1), and electrophoresis was performed for 150 min at 90 V, in a TBE buffer supplemented with 20 mM KCl or LiCl. Oligonucleotides were detected by UV-shadowing at 254 nm with a G:BOX (Syngene). Band quantification was done using the ImageJ software. For detection through dye staining, oligonucleotides were loaded in 15% polyacrylamide gels (acrylamide:bisacrylamide 37.5:1), and electrophoresis was performed for 90 min at 100 V, in a TBE buffer supplemented with KCl or LiCl 20 mM. For NMM and SYBR Safe staining, each gel was stained with 50 μM NMM (Sigma) for 30 min and imaged; after washing with pure water for 15 min, the same gel was stained with 1× SYBR Safe (Thermo Fisher) for 30 min and imaged. For Thioflavin T (ThT) staining, gels were stained with 50 μM ThT (Sigma). Gels were imaged with a ChemiDoc MP Imaging System (Bio-Rad), through the SYBR Safe dye channel (excitation 470 nm, emission 560/50 nm). As DNA ladder, we used Thermo Scientific™ GeneRuler Ultra Low Range DNA Ladder, ready-to-use.

### Nuclear magnetic resonance 

The d(AGGGA)_8_ oligonucleotide was diluted at 60 µM strand concentration in 10 mM cacodylic acid buffer pH 7.2 (pH adjusted with LiOH), containing 50 mM KCl or LiCl, then heated at 90°C for a few minutes and slowly cooled to room temperature. D_2_O (10% of total volume) was added to sample solutions before measurements. For each sample, DSS, dissolved in the solvents used for sample measurements, was used as external chemical shift standard. ^1^H nuclear magnetic resonance (NMR) spectra were acquired at 298 K on an Avance III HD 600 MHz spectrometer (Bruker), equipped with a 5 mm triple resonance TCI cryoprobe (^1^H-^13^C-^15^N) including shielded Zgradients. Excitation sculpting was used for peak solvent suppression (pulse program zgesgp). The number of accumulated scans was 16,384 for the the KCl sample and 14,319 for the LiCl sample.

## Results

### Rationale and design of the sequences and conditions

In order to gain a comprehensive understanding of the structures formed by CANVAS-associated repeats, we analysed DNA and RNA sequences composed of an increasing number of the pathogenic AGGGA and AGGAC motifs, as well as the nonpathogenic AGAAA motif. To assess whether the folding behaviour of dAGGGA repeats was unique or shared with other dWGGGW motifs, we also investigated sequences comprising an increasing number of dTGGGT, dTGGGA, and dAGGGT repeats.

We studied sequences composed of *n *= 4, 8, 12, 16 repeats, which can in principle fold intramolecularly into 1, 2, 3, 4 contiguous G4 units, respectively, and set the strand concentrations to 24/*n* μM (i.e. 6 μM for *n *= 4, 3 μM for *n *= 8, 2 μM for *n *= 12 and 1.5 μM for *n *= 16). For all the WGGGW sequences, these strand concentrations correspond to a concentration of 6 μM in potential G4 units (if contiguous G4 units are formed) and, more generally, to 18 μM in potential G-tetrads (irrespective of whether the G4 structures formed are intra- or intermolecular). This choice of concentrations allows for direct comparison of the amplitude of TDS, UV-melting transitions, and CD spectra. Similarly, for PAGE assays, we set strand concentrations to 240/*n* μM for detection by UV-shadowing or at 24/*n* μM for detection by dye staining.

Considering that the stability of G4 structures depends on the type of cation present in the solution (e.g. K⁺ strongly stabilizes G4 structures, whereas Li⁺ does not), and that duplexes are only minimally affected by the type of monocation in the solution, we assessed the potential of pentanucleotide repeats to fold into G4 and into non-G4 structures by working in K⁺ and Li⁺ solutions, respectively.

We used the hexameric d(TGGGTT)_4,8,12,16_ repeats as reference oligonucleotides forming “well-behaved” G-quadruplexes that have been previously studied [35–37]. The TDS, CD, and melting profiles of these sequences in KCl indicate that these oligonucleotides fold into intramolecular structures composed of 1, 2, 3, and 4 similar and noninteracting contiguous G4 units of hybrid conformation (Supplementary Fig. S1), similarly to GGGTTA telomeric repeats [38].

### A first assessment of the G4 folding potential of WGGGW repeats

As a first assessment of the G4 folding potential of WGGGW repeats, we carried out two simple and straightforward assays: an iso-FRET and an NMM assay. These isothermal assays, in a 96-well plate format, allow a rapid screening of the propensity of oligonucleotides to fold into G4s. In the iso-FRET competition assay [32], an RNA G4 coupled to a quencher (37Q) is mixed with the oligonucleotide whose structure is to be tested (the “competitor”) and with a G4 ligand. A FAM-labelled oligonucleotide (F22) complementary to 37Q is then added. If the competitor does not form a G4, the G4 ligand will bind to the 37Q G4, stabilizing its structure and preventing its hybridization with F22; hence, FAM fluorescence will not be quenched. If the competitor forms a G4, the G4 ligand will bind to it rather than to 37Q, allowing the hybridization of F22 to 37Q; this will lead to the quenching of FAM fluorescence. The NMM assay is based on the enhancement of fluorescence emission of the G4 ligand NMM upon binding G4 [39]: NMM is added to a solution containing the oligonucleotide whose structure is to be tested; if the oligonucleotide folds into a G4, NMM binds to it and its fluorescence increases.

**Figure 1.**
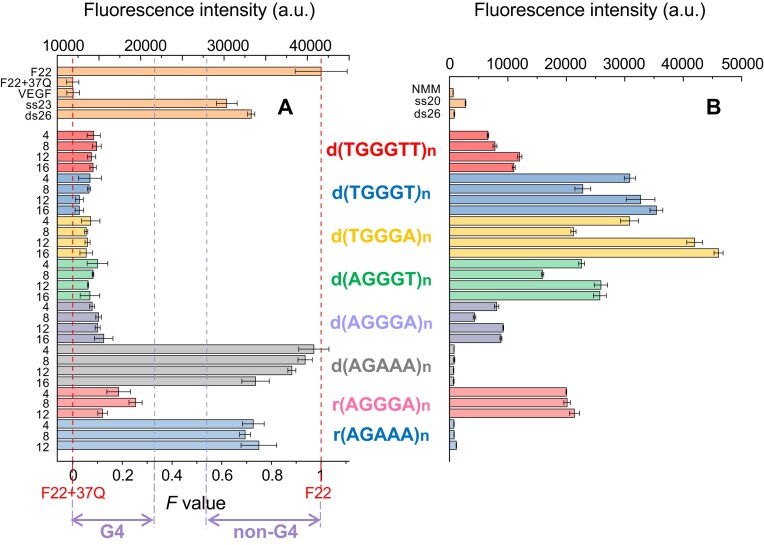
Iso-FRET and NMM assays. (**A**) Iso-FRET assay with pentanucleotide repeats as competitors of the 37Q oligonucleotide in a solution containing 20 mM KCl and 80 mM LiCl, at 20°C. A low fluorescence *F* value (*F* < 0.33) indicates that at least a fraction of the pentanucleotide repeat is folded into G4s, while a high fluorescence *F* value (*F* > 0.54) indicates that the pentanucleotide repeat is not folded into G4s. (**B**) NMM assay on pentanucleotide repeats in 100 mM KCl, at 20°C. An increase in NMM fluorescence relative to the NMM alone or in the presence of single-stranded (ss20) and duplex (ds26) DNA controls indicates that a fraction of the pentanucleotide is folded into G4s. Buffer: 10 mM cacodylic acid, pH 7.2.

Both iso-FRET and NMM assays indicated that all (WGGGW)_n_ oligonucleotides formed G4s in the presence of KCl, unlike the nonpathogenic CANVAS dAGAAA and rAGAAA repeats (Fig. 1). In the NMM assay, the presence of G4s was indicated by the several-fold increase in NMM fluorescence in 100 mM KCl (ranging from 5-fold for dAGGGA repeats to 23-fold for dTGGGA repeats; Fig. 1), along with the decrease in fluorescence as the KCl concentration was progressively reduced while keeping the ionic strength constant (Supplementary Fig. S2). These two assays allow a rapid assessment of the presence of G4s in solution, but they do not allow to determine whether G4s are the only structures present in solution or whether they are in equilibrium with non-G4 structures.

In the following subsections, we present the UV-spectroscopy and PAGE characterization of each repeat type separately, starting with the simplest case (dTGGGT repeats) and concluding with the pathogenic CANVAS repeats.

### dTGGGT repeats

**Figure 2.**
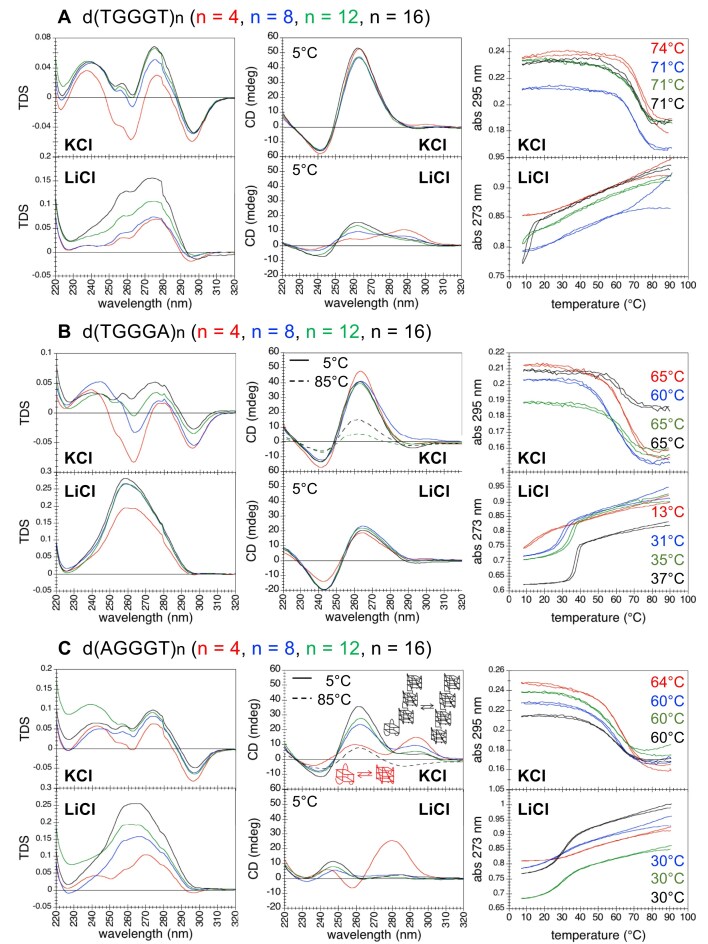
UV-spectroscopy investigation of dWGGGW repeats. TDS, CD spectra and absorbance as a function of temperature (cooling and heating curves) of (**A**) d(TGGGT)_n_, (**B**) d(TGGGA)_n_, and (**C**) d(AGGGT)_n_ (red: *n* = 4, blue: *n* = 8, green: *n* = 12, black: *n* = 16) in 100 mM KCl or LiCl, at strand concentrations of 24/*n* μM (corresponding to 6 μM of potential G4 units or 18 μM of potential G-tetrads). *T*m values are reported. For CD spectra at 85°C, only those that display a relatively strong signal at this temperature are shown (dashed lines). All CD spectra and normalized melting curves are reported in Supplementary Fig. S3. Buffer: 10 mM cacodylic acid, pH 7.2.

**Figure 3.**
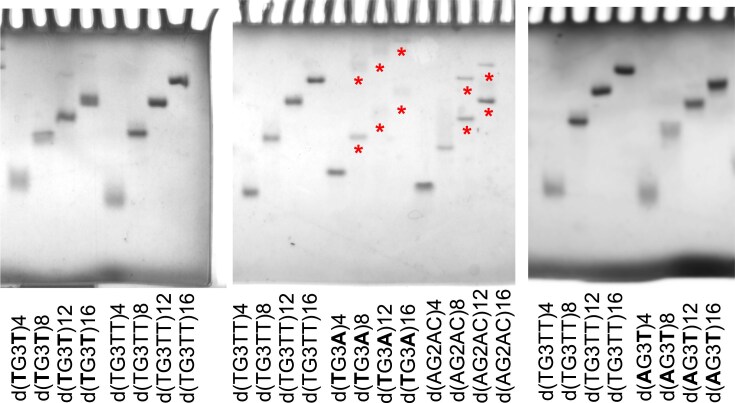
PAGE investigation of dWGGGW and dAGGAC repeats. Migration pattern of DNA pentanucleotide repeats and of the reference oligonucleotides d(TGGGTT)_n_ in 100 mM KCl, detected by UV-shadowing, at strand concentrations of 240/*n* μM. The ”*” symbols mark the fast and the slow migrating bands detected for dTGGGA and dAGGAC repeats. Sample buffer: 10 mM cacodylic acid, pH 7.2. Electrophoresis was carried out in a cold room.

In **KCl**, dTGGGT repeats displayed TDS characteristic of G4s and CD spectra characteristic of parallel G4s, with relatively high melting temperatures (74°C for *n* = 4 and 71°C for *n* = 8, 12, 16; Fig. 2A). The structures formed by the four oligonucleotides were completely unfolded at high temperature, as assessed by CD spectra at 85°C (the CD spectra at 5°C and at 85°C and the normalized melting curves of all dWGGGW repeats are shown in Supplementary Fig. S3). In a PAGE assay, d(TGGGT)_n_ migrated as single bands, displaying a mobility indicative of intramolecular folding (Fig. 3) when compared to the mobility of the G4-forming reference oligonucleotides d(TGGGTT)_n_.

The TDS, CD spectra and melting curves of dTGGGT oligonucleotides comprising 8, 12, and 16 repeats were nearly identical; these features, along with the intramolecular nature of the formed structures as assessed by PAGE, supported the formation of intramolecular structures composed of 2, 3, and 4 contiguous G4 units, as discussed for dTGGGTT repeats (Supplementary Fig. S1) and previously shown for dGGGTTA telomeric repeats [38]. The structure formed by four repeats (allowing the formation of a single G4 unit) exhibited a CD spectrum nearly identical to the CD spectra of the longer repeats, but a different G4 TDS signature and a higher *T*m value (74°C versus 71°C). As for dTGGGTT repeats (Supplementary Fig. S1), the higher *T*m of the single G4 formed by d(TGGGT)_4_ may be explained by stabilizing interactions of the free T nucleotides at the 5′ and 3′ end with the G4 core, which are likely impaired in the context of longer repeats where each internal TT linker is shared by two adjacent G4 units [38].

In **LiCl**, dTGGGT repeats did not form stable structures: they exhibited weak CD signatures at low temperature (5°C) that could be reminiscent of G4s; however, the melting curves of d(TGGGT)_4,8,12_ showed no transition, while the d(TGGGT)_16_ melting curve suggested the formation of a structure with a *T*m below 7°C (the lower limit of the temperature range of our measurements; Fig. 2A).

In conclusion, analysis of dTGGGT repeats revealed a relatively simple and expected behaviour, with folding into intramolecular G4s in KCl and no structuration in LiCl, close to what was observed with the hexameric dTGGGTT motif.

### dTGGGA repeats

As dTGGGT repeats, dTGGGA repeats in **KCl** also displayed TDS characteristic of G4s and CD spectra characteristic of parallel G4, with melting temperatures of 65°C for *n* = 4, 12, 16, and 60°C for *n* = 8 (Fig. 2B). The *T*m values were a few degrees lower than those of dTGGGT repeats. Yet, a fraction of the G4 structures formed by 12 and 16 dTGGGA repeats persisted at high temperatures, as assessed by CD spectra at 85°C (Fig. 2B). The persistence of a folded G4 fraction at high temperature explains the smaller amplitude of the melting transition observed for d(TGGGA)_12_ and d(TGGGA)_16_ (about 0.03 and 0.02, respectively) compared to d(TGGGA)_4_ and d(TGGGA)_8_ (about 0.05; Fig. 2B), since the G4 fraction that remains folded at high temperature does not contribute to the amplitude of the transition. In a PAGE assay, each d(TGGGA)_8,12,16_ oligonucleotide migrated as two bands, one migrating with a mobility comparable to that of the corresponding reference oligonucleotide d(TGGGTT)_n_, and one migrating more slowly, in agreement with the formation of both intra- and intermolecular structures (Fig. 3). Overall, spectroscopic and PAGE data support the formation of intramolecular G4s by d(TGGGA)_4_ and of both intra- and intermolecular G4s by the longer d(TGGGA)_8,12,16_ oligonucleotides, all of which were characterized by a parallel G4 CD signature.

In **LiCl**, dTGGGA repeats did not exhibit TDS and CD spectra characteristic of G4s, nevertheless the melting curves indicated the formation of structures with a *T*m value that increased as the number *n* of repeats increased, from 13°C for *n* = 4 to 37°C for *n* = 16 (Fig. 2B).

In conclusion, dTGGGA repeats fold into intramolecular and intermolecular G4s in KCl and into less stable non-G4 structures in LiCl.

### dAGGGT repeats

In **KCl**, dAGGGT repeats exhibited TDS characteristic of G4 structures, with melting temperatures of 64°C for *n* = 4 and 60°C for *n* = 8, 12, 16 (Fig. 2C). Among these structures, only the one formed by sixteen dAGGGT repeats partially persisted at high temperatures, as assessed by CD spectra at 85°C (Fig. 2C). In a PAGE assay, the migration pattern of dAGGGT repeats, compared to the migration pattern of the reference dTGGGTT repeats, was consistent with the formation of intramolecular structures (Fig. 3).

Unlike dTGGGT and dTGGGA repeats, the shape and intensity of dAGGGT CD spectra depended on the number *n* of repeats: with increasing *n*, the intensity of the band at 262 nm increased whereas the intensity of the band at 292 nm decreased (Fig. 2C). dAGGGT is the telomeric motif of *Giardia duodenalis*, a flagellated parasitic protist. An NMR study of the structure of four dAGGGT repeats [more precisely of the oligonucleotide dT(AGGGT)_3_GGG)] has shown that this sequence folds into two distinct intramolecular G4 structures in K^+^ solutions: a basket-type antiparallel G4 with two G-tetrads and a parallel G4 with three G-tetrads, schematically illustrated in Fig. 2C, which are characterized by distinct CD spectra [40]. The d(AGGGT)_4_ CD spectrum we obtained in KCl was consistent with the coexistence of these two G4 structures. In order to interpret the evolution of the CD spectra of d(AGGGT)_n_ with increasing *n*, we considered the hypothesis that d(AGGGT)_8,12,16_ could fold into intramolecular structures where one of the G4 units was in equilibrium between the antiparallel and the parallel conformation (as reported for the single G4 formed by four dAGGGT repeats), whereas the other G4 units adopted a parallel conformation, as schematically illustrated in Fig. 2C for *n* = 16. To test this hypothesis, we calculated the CD spectra of hypothetical structures composed of *m* G4 units (*m *= 2, 3, 4): one unit with the CD spectrum of the d(AGGGT)_4_ oligonucleotide (i.e. corresponding to an equilibrium between the parallel and the antiparallel conformations), and *m*-1 G4 units with the CD spectrum of a parallel G4. The calculated spectra reproduced the experimental CD spectra of 8, 12, and 16 dAGGGT repeats quite satisfactorily (Supplementary Fig. S4), supporting our hypothesis.

In **LiCl**, d(AGGGT)_8,12,16_ did not exhibit TDS and CD spectra characteristic of G4s. As for dTGGGA repeats, their melting profiles showed transitions indicative of the formation of structures with a *T*m around 30°C (Fig. 2C). However, the temperature ranges over which the melting transition of d(AGGGT)_8,12,16_ occurred were much broader than those of dTGGGA repeats. For example, the melting transition of d(TGGGA)_16_ occurred within a 10°C range (from 42°C to 32°C; Fig. 2B), whereas that of d(AGGGT)_16_ occurred over a 40°C range (from 55°C to 15°C; Fig. 2C). Furthermore, unlike the *T*m of dTGGGA repeats, which increased with the number *n* of repeats, the *T*m of dAGGGT repeats was independent of *n*. These features suggest that the non-G4 structures formed by dTGGGA and dAGGGT repeats have different structural and thermodynamic characteristics.

The TDS and the CD spectrum of d(AGGGT)_4_ in LiCl were quite different from those of the longer repeats. d(AGGGT)_4_ melting curves did not show a clear transition at 260 and 273 nm (Fig. 2C), while they showed a transition around 40°C at 295 and 245 nm (Supplementary Fig. S5). Although the TDS of d(AGGGT)_4_ may be reminiscent of G4 structures, the CD spectrum (with a minimum at 258 nm and a maximum at 280 nm) is not characteristic of G4s (e.g. antiparallel G4 have a minimum around 265 nm and a maximum around 295 nm); furthermore, as for the longer repeats, the NMM assay did not provide evidence of the presence of d(AGGGT)_4_ G4 structures in LiCl (Supplementary Fig. S2).

In conclusion, dAGGGT repeats fold into intramolecular G4s in KCl and into non-G4 structures in LiCl, which are less stable than the non-G4 structures formed by dTGGGA repeats.

### CANVAS pathogenic dAGGGA repeats

**Figure 4.**
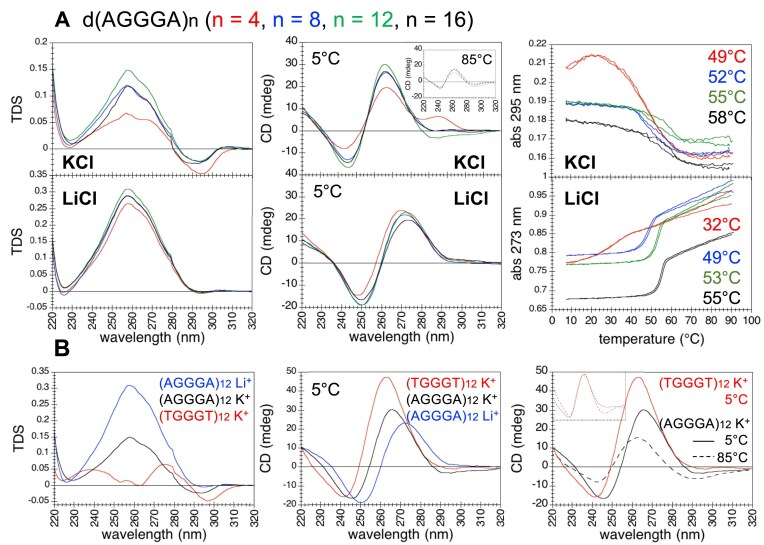
UV-spectroscopy investigation of CANVAS pathogenic dAGGGA repeats. (**A**) TDS, CD spectra, and absorbance as a function of temperature (cooling and heating curves) of d(AGGGA)_n_ (red: *n* = 4, blue: *n* = 8, green: *n* = 12, black: *n* = 16) in 100 mM KCl or LiCl, at strand concentrations of 24/*n* μM (corresponding to 6 μM of potential G4 units or 18 μM of potential G-tetrads). *T*m values of d(AGGGA)_8,12,16_ in KCl are the midpoint values between *T*m^cooling^ and *T*m^heating^. (**B**) TDS and CD spectra of d(AGGGA)_12_ in 100 mM LiCl (blue) or in 100 mM KCl (black), and of d(TGGGT)_12_ in 100 mM KCl (red), at a strand concentration of 2 μM. The normalized CD spectra of d(TGGGT)_12_ at 5°C (red line) and d(AGGGA)_12_ at 85°C (black dashed line) are shown in the inset. Buffer: 10 mM cacodylic acid, pH 7.2.

At first sight, in **KCl**, dAGGGA repeats exhibited TDS, CD spectra and melting profiles reminiscent of G4s, but challenging to interpret (Fig. 4A). Unlike the other dWGGGW repeats, the melting curves of d(AGGGA)_8,12,16_ exhibited hysteresis (i.e. the cooling and the heating profiles were not identical), and their midpoint melting temperatures—defined as *(T*m^cooling^* + T*m^heating^*)/2*—increased with the number of repeats. Melting experiments performed on d(AGGGA)_4,5,8,12,16,20_ repeats at a higher rate of temperature variation confirmed an increase in midpoint melting temperatures and hysteresis with increasing repeat number (Supplementary Fig. S6). CD spectra at 85°C indicated that a fraction of d(AGGGA)_8,12,16_ oligonucleotides remained folded at high temperature (Fig. 4A); this explains the small amplitude (about 0.02) of their melting transitions at 295 nm (Fig. 4A), as discussed above for dTGGGA repeats. To gain insight into the structures formed in KCl, we carried out experiments in LiCl.

Experiments in **LiCl** provided evidence of the formation of non-G4 structures with *T*m values than increased with increasing the number *n* of repeats: from 32°C for *n *= 4 to 49°C, 53°C and 55°C for *n *= 8, 12, and 16, respectively (Fig. 4A). These *T*m values were higher than those of dTGGGA and dAGGGT repeats in LiCl and comparable to the *T*m values determined at 295 nm in KCl. We therefore wondered whether these non-G4 structures could be present in KCl solutions in equilibrium with the G4 structures.

A comparison with the TDS and CD spectra of dTGGGT repeats helped us to correctly interpret the TDS and CD spectra of dAGGGA repeats in KCl. Indeed, TDS and CD spectra of d(AGGGA)_8,12,16_ in KCl had shapes that were intermediate between those of dTGGGT repeats in KCl (folding into parallel G4s) and dAGGGA repeats in LiCl (folding into a non-G4 structure; Fig. 4B). These features suggested that, in KCl solution, d(AGGGA)_8,12,16_ folded into both G4 and non-G4 structures that coexisted in equilibrium. Furthermore, the CD spectra of dAGGGA repeats in KCl at high temperature (85°C) were identical in shape to the CD spectra of dTGGGT repeats in KCl at low temperature (5°C; Fig. 4B), indicating that the dAGGGA structures that persisted in KCl at high temperature were the G4s. This was consistent with the results obtained in LiCl, which showed that the non-G4 structures were completely unfolded at high temperature (Fig. 4A).

**Figure 5.**
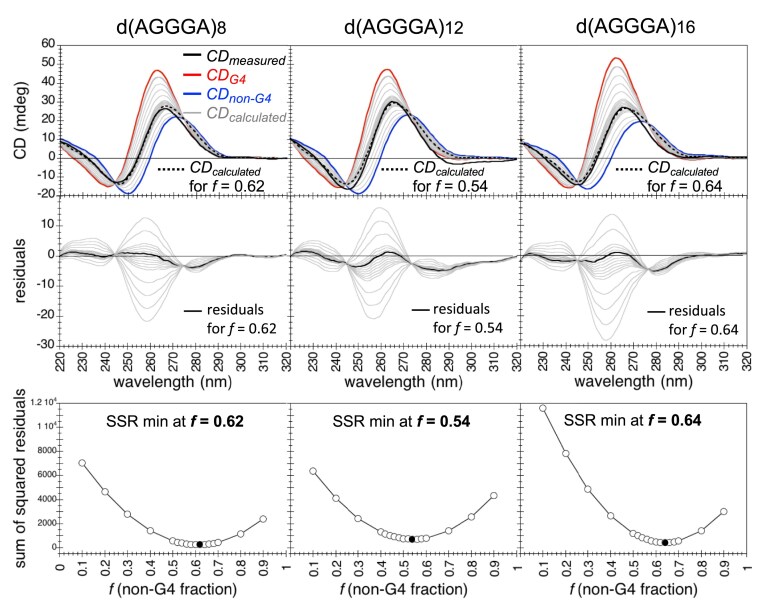
Estimation of the non-G4 fraction *f* and G4 fraction (1 − *f *) of d(AGGGT)_8,12,16_ in KCl. The CD spectra of d(AGGGA)_n_ (24/*n* μM) in 100 mM KCl (black bold line), denoted CD_measured_, result from the coexistence of a non-G4 structure and a G4 structure. The non-G4 fraction *f* and the G4 fraction (1 − *f *) have been estimated as follows. We assumed that: (i) the CD spectrum of d(TGGGT)_n_ (24/*n* μM) in 100 mM KCl (red bold line), denoted CD_G4_, corresponds to the CD spectrum of the G4 structure of d(AGGGA)_n_ present in KCl; (ii) the CD spectrum of d(AGGGA)_n_ (24/*n* μM) in 100 mM LiCl (blue bold line), denoted CD_non-G4_, corresponds to the CD spectrum of the non-G4 structure of d(AGGGA)_n_ present in KCl. Under these assumptions, the non-G4 fraction *f* was determined as the value that provides the best approximation of CD_measured_ as a weighted sum of the two reference spectra: CD_measured_ ≈ *f* CD_non-G4_ + (1 − *f *) CD_G4_. Calculated spectra CD_calculated_(*f *) = *f* CD_non-G4_ + (1 − *f *) CD_G4_ were generated for *f* values ranging from 0.1 to 0.9 (grey lines). The best *f* value was determined by minimization of the sum of squared residuals (SSR): SSR(*f *) = Σ_i_ [CD_measured_(λ_i_) − CD_calculated_(*f , *λ_i_)]^2^. For each repeat, the best *f* value and its corresponding calculated spectrum (dotted black bold line) are reported.

Through an analysis of the CD spectra of d(AGGGA)_8,12,16_ in KCl in terms of the CD spectra of dTGGGT repeats in KCl (pure G4s) and of dAGGGA repeats in LiCl (pure non-G4s), we could estimate that the percentage of d(AGGGA)_8,12,16_ folded into non-G4 structures in 100 mM KCl amounted to 50%–60% (Fig. 5). Decreasing the KCl concentration to 30, 10 or 3 mM resulted in a gradual shift of the TDS and CD spectra towards the non-G4 signature, indicating that the equilibrium between G4 and non-G4 structures is modulated by KCl concentration (Supplementary Fig. S7).

PAGE assays confirmed that, in KCl, d(AGGGA)_8,12,16_ existed in equilibrium between G4 and non-G4 structures, provided information on the molecularity of these structures, and yielded insights into the structures formed by the shortest oligonucleotide d(AGGGA)_4_.

**Figure 6.**
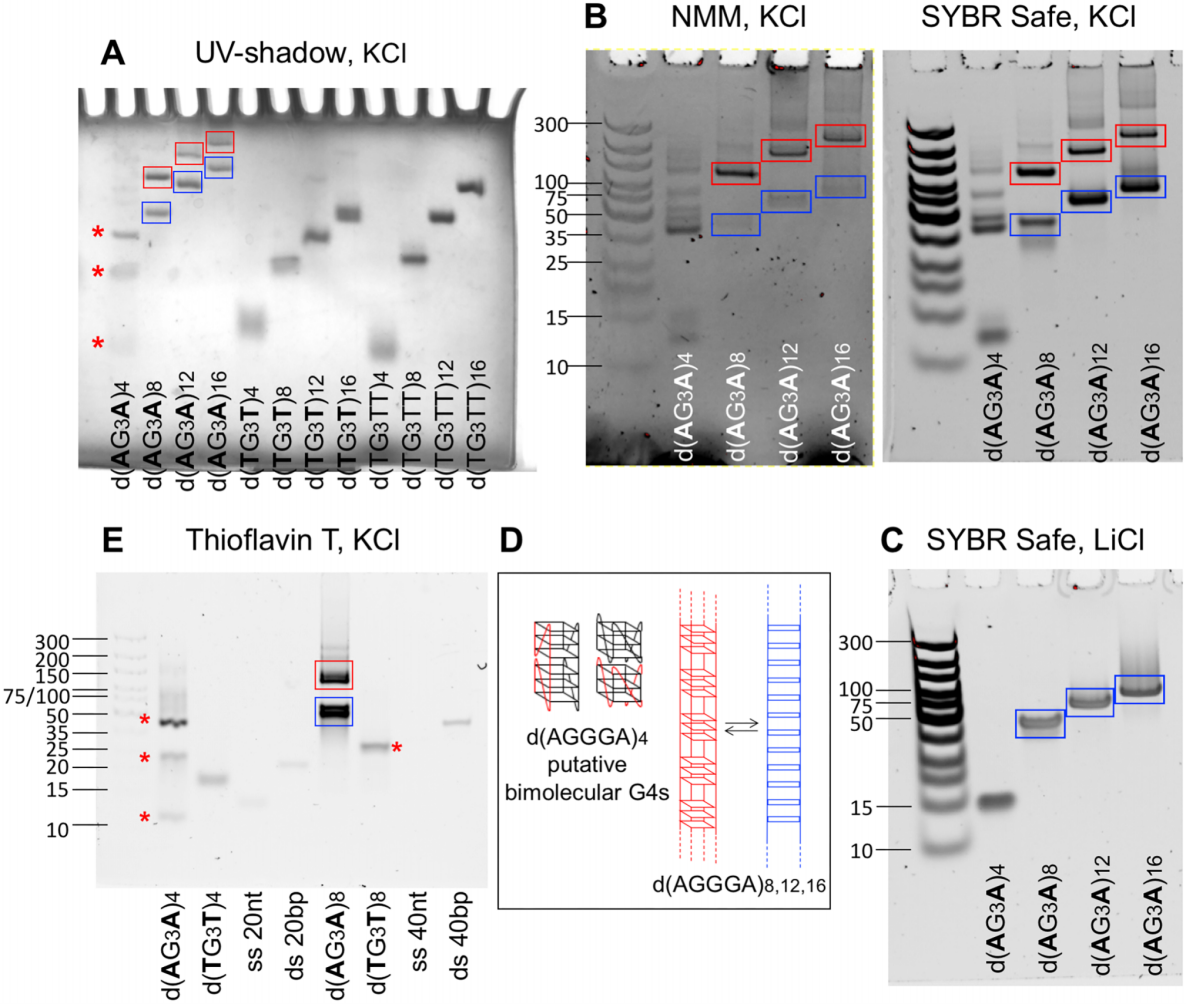
PAGE investigation of CANVAS pathogenic dAGGGA repeats. Migration pattern of dAGGGA repeats and of the reference oligonucleotides d(TGGGTT)_n_ and d(TGGGT)_n_ (**A**) in 100 mM KCl, detected by UV-shadowing; (**B**) in 100 mM KCl, detected by NMM and SYBR Safe staining; (**C**) in 100 mM LiCl, detected by SYBR Safe staining; (**E**) in 100 mM KCl, detected by ThT staining. Strand concentrations: 240/*n* μM in gel (**A**), 24/*n* μM in gels (**B, C, E**). Electrophoresis of gel (**A**) was carried out at room temperature, whereas gels (**B, C, E**) were run in a cold room. The symbol ”*” marks the major bands detected for d(AGGGA)_4_ in gels (**A**) and (**E**) and the d(TGGGT)_8_ band in gel (**E**). DNA ladder sizes: 10, 15, 20, 25, 35, 50, 75, 100, 150, 200, 300 bp. Sample buffer: 10 mM cacodylic acid, pH 7.2. (**D**) Schematic representation of the intermolecular G4 and duplex structures formed by d(AGGGA)_8,12,16_ corresponding to the gel bands framed by red and blue rectangles, respectively, and schematic representation of putative bimolecular G4s that may be formed by d(AGGGA)_4_.

In PAGE assays in KCl, two bands were detected for each of the **d(AGGGA)_8,12,16_** oligonucleotides. These bands migrated more slowly than the band detected for the corresponding reference oligonucleotide d(TGGGTT)_n_ and even more slowly than the d(TGGGT)_16_ band, supporting the formation of at least two distinct intermolecular structures (Fig. 6A).

When compared to the DNA size markers, the relative mobilities of the d(AGGGA)_8,12,16_ faster bands in KCl (Fig. 6B) were similar to the relative mobilities of the d(AGGGA)_8,12,16_ single bands detected in LiCl (Fig. 6C): the faster band of d(AGGGA)_8_ migrated just below the 50 bp band of the DNA ladder, the faster band of d(AGGGA)_12_ just below the 75 bp marker, and the faster band of d(AGGGA)_16_ just below the 100 bp band. These findings support the conclusion that the d(AGGGA)_8,12,16_ faster bands in KCl correspond to the non-G4 structures. Although caution should be exercised when drawing conclusions based on gel mobility, the mobility of the d(AGGGA)_8,12,16_ faster bands was consistent with the formation of 40, 60, and 80 bp duplexes, respectively, when compared to the DNA size markers.

Staining the gel with the G4-specific fluorescent probe NMM resulted in a stronger NMM fluorescence intensity in the slower migrating bands than in the faster ones, confirming that the d(AGGGA)_8,12,16_ slower bands in KCl corresponded to the G4 structures (Fig. 6B).

Unlike UV-shadowing detection, gel staining with fluorescent DNA probes (such as SYBR and NMM) does not allow a relative quantification of the DNA in each band because the quantum yield of a fluorescent DNA probe may depend on the DNA structure. When revealed by UV-shadowing, the fast and the slow bands of each d(AGGGA)_8,12,16_ oligonucleotide had comparable intensities (Fig. 6A), corresponding to a G4/non-G4 ratio of ≈ 0.5/0.5 for d(AGGGA)_8,16_ and 0.4/0.6 for d(AGGGA)_12_ in 100 mM KCl. This estimation of the G4 and non-G4 fractions was consistent with the one deduced from the analysis of CD spectra (Fig. 5).

Overall, the results of PAGE assays in Fig. 6A–C support that d(AGGGA)_8,12,16_ oligonucleotides are in equilibrium between an intermolecular G4 structure and an intermolecular duplex structure (Fig. 6D).

The migration patterns of **d(AGGGA)_4_** were more complex, displaying multiple bands. We will focus on the major bands detected by four different methods (UV-shadowing, SYBER Safe, NMM and ThT staining). UV-shadowing revealed at least three bands (Fig. 6A). The two most intense bands detected by SYBR Safe staining migrated between the 10 and 15 bp markers (fastest band) and between the 35 and 50 bp markers (slower band); these bands were also detected by NMM staining (Fig. 6B). Staining with ThT revealed three bands: the two bands also revealed by SYBR Safe and NMM staining (one between the 10 and 15 bp markers, the other between the 35 and 50 bp markers) and a third band between the 20 and 25 bp markers; this third band migrated as d(TGGGT)8 (Fig. 6E). This observation was consistent with the migration behaviour of the d(AGGGA)_4_ intermediate band detected by UV-shadowing, which was comparable to that of d(TGGGT)_8_ (Fig. 6A). All these d(AGGGA)_4_ bands should correspond to G4 structures, since they were not detected in LiCl, where a single band was detected migrating as the 15 bp marker (Fig. 6C). Based on these results, we propose the following interpretation: the fast migrating band of d(AGGGA)_4_ in KCl (the one between 10 and 15 bp) may correspond to an intramolecular G4; the intermediate band (the one between 20 and 25 bp), which migrates the same distance of the intramolecular structures formed by d(TGGGT)_8_, may correspond to a bimolecular G4 formed by the stacking of two parallel G4 units (two single G4 units or two interstrand G4 units, Fig. 6D); the slow migrating band (the one between 35 and 50 bp) may correspond to a tetramolecular G4.

ThT staining of both d(AGGGA)_8_ bands in KCl (Fig. 6E) is not surprising. Indeed ThT, known to be a fluorescent probe for G4 structures [41], has also been shown to bind to parallel stranded DNA duplexes containing GA motifs, enhancing its fluorescence, and an intercalation mode between a G:G and an A:A base-pair has been proposed [42].

Given that **d(AGGGA)_8,12,16_** repeats form (at least) two distinct structures in KCl (G4s and duplexes), we wondered whether these structures interconverted and how fast the duplex/G4 equilibrium is reached. To provide some clues, we tested whether a preformed duplex structure would convert into a G4 structure to reach the duplex/G4 equilibrium or whether the duplex remained in a kinetically trapped state. To answer to this question, we annealed dAGGGA repeats in a LiCl solution and then added KCl. The CD spectra evolved over time towards those of dAGGGA repeats in KCl solutions; however, the structural conversion was slow, taking several hours at 5°C (Supplementary Fig. S8). Unfortunately, the reverse experiment (starting from a pure G4) is more difficult to perform, since this would require purifying the G4 structure, that, even at high KCl concentration, is in equilibrium with the duplex structure (Supplementary Fig. S7).

In conclusion, in KCl, the d(AGGGA)_8,12,16_ oligonucleotides are in equilibrium between intermolecular G4s and noncanonical duplexes, whereas d(AGGGA)_4_ is likely in equilibrium between polymorphic G4 structures of different molecularities. In LiCl, all the dAGGGA repeats folded into duplexes more stable than the non-G4 structures formed by dTGGGA and dAGGGT repeats.

Although UV-spectroscopy and PAGE provided solid evidence of an equilibrium between G4s and duplexes for d(AGGGA)_8,12,16_ oligonucleotides, we wondered whether NMR spectroscopy could provide additional evidence, as previously found for other unstable repeats [43]. The ^1^H NMR spectrum of d(AGGGA)_8_ in KCl solution (where the oligonucleotide adopts both G4 and duplex conformations) displayed peaks in the 10–12 ppm region characteristic of guanine imino protons (Supplementary Fig. S9A). In contrast, the ^1^H NMR spectrum of d(AGGGA)_8_ in LiCl solution (where only duplex structures are formed) showed no detectable signals in this region or further downfield (a very weak signal was detected upfield, around 9.5 ppm; Supplementary Fig. S9B). This absence of imino signals made it challenging to highlight the equilibrium between G4 and duplexes by ^1^H NMR; nevertheless, it offered some insight into the nature of base pairing in the duplex form of AGGGA repeats: it might indicate that the duplex form mainly relies on base pairing involving only amino protons (Supplementary Fig. S9B).

### CANVAS pathogenic dAGGAC repeats and nonpathogenic dAGAAA repeats

So far, all the repeats investigated belonged to the WGGGW family, where W is A or T. As CANVAS also involves other types of repeats, we also studied the dAGGAC motif (the second pathogenic motif identified in CANVAS patients) and repeats of the nonpathogenic dAGAAA motif (which, in most individuals, are limited to eleven repeats).

**Figure 7.**
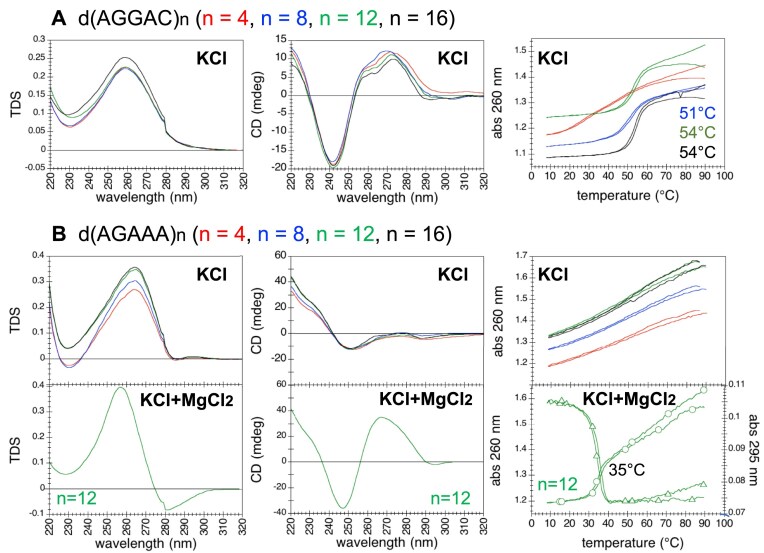
UV-spectroscopy investigation of CANVAS pathogenic dAGGAC and nonpathogenic dAGAAA repeats. TDS, CD spectra and absorbance as a function of temperature (cooling and heating curves) of (**A**) d(AGGAC)_n_ and (**B**, top panel) d(AGAAA)_n_ (red: *n* = 4, blue: *n* = 8, green: *n* = 12, black: *n* = 16) in 100 mM KCl, at strand concentrations of 24/*n* μM (corresponding to 6 μM of potential G4 units or 18 μM of potential G-tetrads). (**B**, bottom panel) TDS, CD spectrum and absorbance at 260 nm (circles) and 295 nm (triangles) as a function of temperature (cooling and heating curves) of 2 μM d(AGAAA)_12_ in a solution containing 100 mM KCl and 10 mM MgCl_2_. Buffer: 10 mM cacodylic acid, pH 7.2. *T*m values are reported.

Although **dAGGAC** repeats might potentially fold into G4s in KCl due to the presence of two consecutive guanines, their TDS and CD spectra clearly indicated the formation of non-G4 structures by d(AGGAC)_8,12,16_, with *T*m values close to those of the duplex structures formed by dAGGGA repeats (Fig. 7A). The CD spectrum (recorded at 5°C) suggested that the shorter oligonucleotide d(AGGAC)_4_ was likely folded at low temperature; yet, and unexpectedly, the melting profiles (at the different wavelength of measurements) showed no transition, indicating that the structure was either not stable or weakly enthalpy-dependent [44] (Fig. 7A). Very similar TDS, CD spectra and melting curves were obtained in LiCl (data not shown). In a PAGE assay, the longer repeats d(AGGAC)_12_ and d(AGGAC)_16_ migrated as two bands, supporting the formation of two distinct structures (Fig. 3).

CD spectra and melting curves of **dAGAAA** repeats in KCl supported the conclusion that these nonpathogenic repeats did not fold into any stable structure (Fig. 7B). However, the addition of 10 mM MgCl_2_ induced the formation of a structure, as evidenced by the CD spectrum and the steep transition in the melting curves, with a *T*m value of 35°C (Fig. 7B).

### RNA sequences: CANVAS rAGGGA, rAGGAC, and rAGAAA repeats

As CANVAS repeats are transcribed, RNA motifs are also relevant. While dAGGGA repeats folded into both intramolecular G4s and duplexes in KCl, **rAGGGA** repeats only folded into parallel G4s as indicated by TDS and CD spectra (Supplementary Fig. S10), with *T*m values around 83°C. rAGGGA repeats, folding exclusively into G4 structures, are therefore less polymorphic than their DNA counterparts. In a PAGE assay, both r(AGGGA)_4_ and r(AGGGA)_8_ migrated the same distance as the reference r(GGGUUA)_3_GGG oligonucleotide (Supplementary Fig. S10). RNA GGGUUA repeats (mimicking transcribed telomeric repeats) are prone to folding into parallel G4 units that stack on the top of each other [45–47]. In particular, the r(GGGUUA)_3_GGG oligonucleotide has been shown to form a dimer of two stacked G4s [48]. Taken together, the PAGE migration patterns of rAGGGA repeats, compared to the reference r(GGGUUA)_3_GGG oligonucleotide, suggest that r(AGGGA)_4_ and r(AGGGA)_8_ share a similar structural arrangement composed of two stacked G4 units, differing in molecularity: four repeats form a dimer in which two separate G4s stack upon each other, whereas eight repeats fold intramolecularly into two G4 units that stack in the same manner (Supplementary Fig. S10).

The **rAGGAC** behave similarly to its corresponding DNA motif: TDS, melting profiles indicated that r(AGGAC)_8,12_ folded into non-G4 structures with *T*m values of 46°C and 48°C, respectively; PAGE highlighted the formation of two distinct structures for both oligonucleotides (Supplementary Fig. S10). Although r(AGGAC)_4_ displayed a CD spectrum identical to that of longer repeats, it did not exhibit any melting transition, suggesting the formation of a structure either unstable or weakly enthalpy-dependent, as found for d(AGGAC)_4_ repeats (Supplementary Fig. S10).

Finally, **rAGAAA** repeats exhibited strong CD signals, but no melting transitions (Supplementary Fig. S10), suggesting the formation of an enthropy-driven single-stranded helical conformation, based on the stacking of the purine bases, as documented for poly(A) [49].

### Insights into dTGGGA, dAGGGT and dAGGAC non-G4 structures

Purine-rich oligonucleotides can adopt noncanonical duplex conformations, notably parallel duplexes based on G•G and A•A base pairs, as proposed, for example, for dGA and dGGA repeats [50, 51]. The formation of antiparallel duplexes involving G•A base pairs has also been proposed for dGA repeats [52]. As previously discussed, a PAGE assay showed that the migration patterns of **d(AGGGA)_8,12,16_** oligonucleotides in LiCl, when compared to the migration of DNA size markers, were consistent with the formation of intermolecular duplexes (Fig. 6C).

To gain insight into the nature of the non-G4 structures of the other DNA repeats, we carried out a PAGE assay in LiCl (Supplementary Fig. S11A). Within the limitation of using Watson–Crick duplexes as size markers for noncanonical duplexes, the migration patterns of **dAGGAC** repeats were consistent with the formation of both intra- and intermolecular duplexes, supporting an antiparallel strand orientation, at least for the intramolecular form. In contrast, the migration patterns of **dTGGGA** repeats were consistent with the formation of exclusively intermolecular duplexes (similarly to AGGGA repeats), suggesting a parallel strand orientation.

Although the dAGGGT motif differs from the dTGGGA motif only in the position of the A and T nucleotides, the migration patterns of **dAGGGT** repeats in LiCl differed markedly from those of dTGGGA repeats (Supplementary Fig. S11A), as did their CD spectra and melting profiles (Fig. 2). Specifically, dAGGGT repeats migrated as a single band, faster than the intermolecular structures formed by dTGGGA and dAGGAC repeats, but slower than the intramolecular structures formed dAGGAC repeats (Supplementary Fig. S11A). To gain insight into the molecularity of the structure formed by dAGGGT repeats in LiCl, we assessed whether *T*m depended on oligonucleotide strand concentration. We carried out melting experiments at concentrations spanning one and two orders of magnitude: in contrast to TGGGA repeats, AGGGT repeats showed no increase in melting temperature, suggesting—though not conclusively demonstrating—the formation of an intramolecular structure (Supplementary Fig. S11B). Overall, our data on dAGGGT repeats are not sufficient to propose a structural model for the non-G4 conformation; nevertheless, they highlight that dAGGGT repeats exhibit distinct structural and thermodynamics properties compared to dTGGGA repeats.

## Discussion

**Figure 8.**
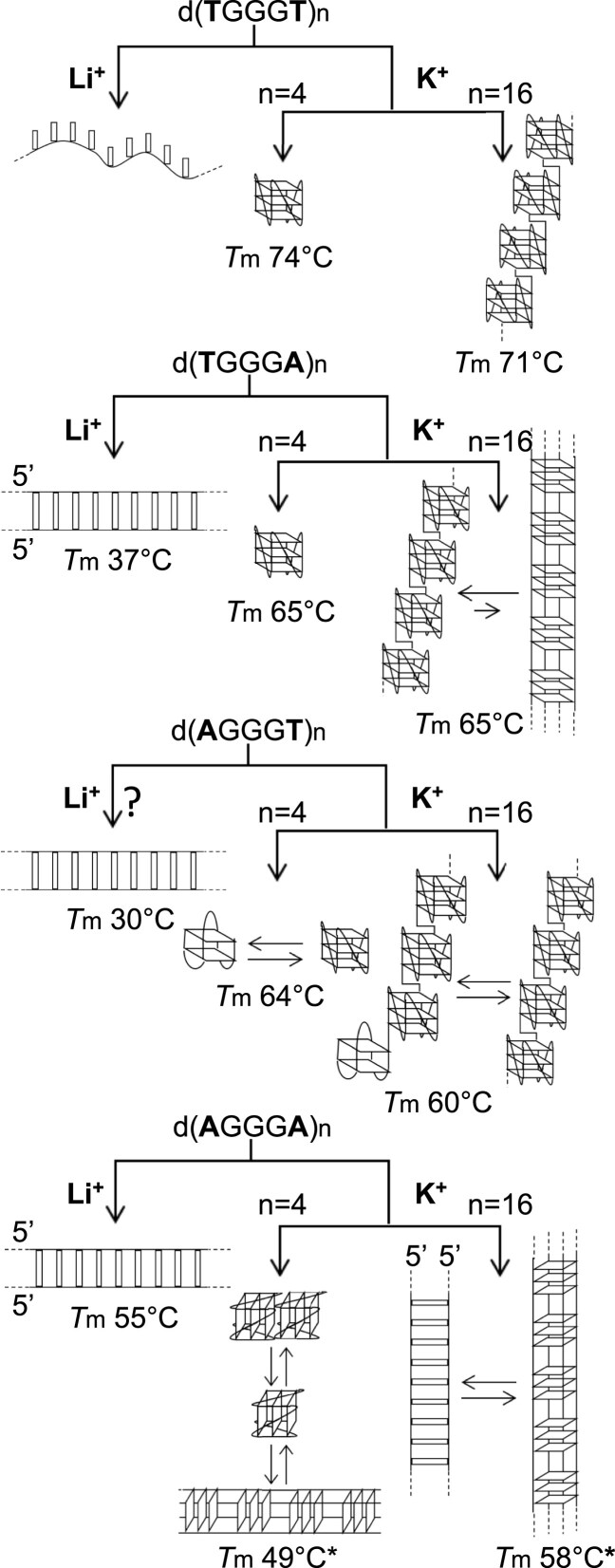
Conformational landscape of dWGGGW repeats. This conformational landscape refers to results obtained in 100 mM LiCl or KCl. The structures shown for *n* = 16 are also representative of the structures formed by 8 and 12 repeats (for which, the intramolecular G4s are composed of two and three G4 units, respectively). dTGGGA and dAGGGA duplexes are likely parallel duplexes. The nature of the dAGGGT non-G4 structure adopted in Li^+^ remains to be determined. *T*m values are those obtained for *n* = 16. The symbol “*” next to *T*m values indicates that a fraction of G4s persists at 85°C.

Previous structural studies of CANVAS-associated repeats have examined sequences comprising a limited number of repeats (typically four or eight for DNA). While all these studies highlighted the G4 folding potential of both DNA and RNA AGGGA repeats, they reported conflicting conclusions regarding the G4 conformation and molecularity. The initial aim of our study was to provide a more comprehensive understanding of the folding behaviour of the pathogenic AGGGA and AGGAC repeats, as well as the nonpathogenic AGAAA repeats, by systematically studying an increasing number of repeats. In early UV-spectroscopy experiments with dAGGGA repeats, we observed features that were highly unusual for G4s. These features suggested a more complex behaviour than expected or previously reported and prompted us to broaden our investigations to include dTGGGT, dTGGGA and dAGGGT repeats and to study the pentanucleotide repeats not only in K^+^ but also in Li^+^ solutions. Unlike K^+^, Li^+^ does not promote G4 formation; however it does not destabilize G4s if other G4-stabilizing cations are present [53]. Analyses of dAGGGA repeats in Li^+^ and of other dWGGGW repeats in K^+^ were critical for interpreting the atypical TDS and CD spectra of dAGGGA repeats observed in K^+^. This expanded approach revealed that, unlike the other dWGGGW repeats, dAGGGA repeats do not exclusively fold into G4s under physiologically relevant K^+^ conditions; moreover, it uncovered the polymorphic conformational landscape of the dWGGGW repeat family. Our study reveals that, within the dWGGGW family, structural polymorphism increases progressively with the number of adenines (0, 1, or 2), as schematically illustrated in Fig. 8.

**dTGGGT** repeats remain unstructured in the absence of a G4-stabilizing cation. In contrast, in the presence of the G4-stabilizing cation K^+^, they fold into intramolecular G4s, likely composed of regular G4 units (Fig. 8).

Unlike dTGGGT repeats, **dTGGGA** and **dAGGGT** repeats can fold into G4 and non-G4 structures, depending on ionic conditions (K^+^ or Li^+^). Nevertheless, the stability of their non-G4 structures (assessed by melting profiles in lithium) is not sufficiently high compared to that of the their G4 structures (assessed by melting profiles in potassium) to compete with G4 formation in potassium (e.g. for d(TGGGA)_16_: *T*m (non-G4) 37°C versus *T*m (G4) 65°C; for d(AGGGT)_16_: *T*m (non-G4) 30°C versus *T*m (G4) 60°C). As a consequence, although dTGGGA and dAGGGT repeats can potentially fold into non-G4 structures, these latter are not formed in potassium (Fig. 8). Subtle differences were noted between dTGGGA and dAGGGT repeats, which differ only in the position of the A and T bases: (i) in K^+^, dTGGGA repeats display a higher degree of polymorphism at the level of the molecularity of the G4 structure, folding not only into intramolecular G4s but also into intermolecular G4s (Fig. 8); (ii) in Li^+^, data suggest structural differences between the non-G4 structure formed by dTGGGA repeats (likely parallel duplexes) and that formed by dAGGGT repeats. The nature of the structure formed by dAGGGT repeats in LiCl remains to be determined; the independence of their *T*m from strand concentration suggests an intramolecular structure, while their independence from the number of repeats raises the possibility that it may consist of regular, repeated structural units. It is worth noting that dAGGGT repeats mimic a biologically relevant sequence: the telomeric G-strand of the flagellated parasitic protist *Giardia duodenalis*. To date, only the structure formed by four dAGGGT repeats in KCl has been characterized, revealing the presence of at least two distinct major G4 conformations [40]. In the present study, we demonstrate that, in KCl, longer dAGGGT repeat sequences fold exclusively into intramolecular G4s, consistent with a beads-on-a-string model in which the G4 units exhibit reduced conformational polymorphism compared with the single G4 formed by four repeats (Fig. 8).

**dAGGGA** repeats, made exclusively of purine bases, display the higher level of structural polymorphism. Like dTGGGA and dAGGGT repeats, dAGGGA repeats can fold into G4 and non-G4 structrures, depending on ionic conditions. As dTGGGA repeats, there is evidence that the non-G4 structures formed by dAGGGA repeats are parallel intermolecular duplexes. Nevertheless, for dAGGGA repeats, the stability of the duplex structures (at least starting from eight repeats) is sufficiently high compared to the stability of the G4 structures [e.g. *T*m (dx) 55°C versus *T*m (G4) 58°C for *n* = 16] to allow the formation of both duplexes and G4s in potassium, leading to an equilibrium between the two structures (Fig. 8). Of note, and unlike the other dWGGGW repeats, the d(AGGGA)_8,12,16_ detected G4 structures were exclusively intermolecular.

The folding behaviour of **d(AGGGA)_4_** differs from that of the longer dAGGGA repeats in at least two aspects: (i) d(AGGGA)_4_ does not form duplexes in potassium, likely because its duplex structure is not stable enough to compete with its G4 structures [*T*m (dx) 32°C versus *T*m (G4) 49°C]; (ii) the G4s formed by four dAGGGA repeats exhibit a higher degree of polymorphism than the G4s formed by longer dAGGGA repeats, likely forming intra-, bi-, and tetramolecular G4s (Fig. 8). This latter aspect may account for the conflicting conclusions reported in the literature about the G4 conformation and molecularity of four dAGGGA repeats.

The evidence of highly stable intermolecular G4s formed by dAGGGA repeats in potassium (a fraction of which persisted even at 85°C) enabled us to explain two of the features of their melting profiles: (i) an increase in the *T*m values at 295 nm with increasing the number of repeats, which can be explained by the intermolecular nature of the G4 formed by dAGGGA repeats; (ii) hysteresis (not identical cooling and heating curves) which can be explained by the slow dissociation kinetics of long intermolecular G4s (Fig. 4A and Supplementary Fig. S6).

It is worth noting that, in KCl, while d(AGGGA)_8,12,16_ melting curves exhibited hysteresis (Fig. 4A), those of d(AGGGA)_4_ and d(TGGGA)_8,12,16_ did not (Fig. 2B), despite the presence of intermolecular G4 species. While d(AGGGA)_8,12,16_ form exclusively intermolecular structures, d(AGGGA)_4_ and d(TGGGA)_8,12,16_ form both intra- and intermolecular G4s. The absence of hysteresis suggests that the major contribution to their melting curves arises from the intramolecular fraction. This may be the results of two nonexclusive possibilities: the intermolecular species persist at high temperatures [as is likely for d(AGGGA)_4_ and d(TGGGA)_12,16_], or they constitute only a minor fraction [as is likely for d(TGGGA)_8_].

For simplicity, we have discussed the relative stability of the formed structures in terms of their *T*m values. However, caution is warranted when using *T*m values as the sole indicator of structural stability [33]. We highlight two examples to illustrate this point:

1) The non-G4 structures formed by eight dTGGGA and eight dAGGGT repeats exhibit similar *T*m values (31°C and 30°C, respectively), yet their melting profiles differ significantly (Fig. 2). The dTGGGA duplex undergoes a sharper melting transition compared to the dAGGGT structure, indicating differences in folding energetics (enthalpic and entropic contributions).
2) Among all dWGGGW G4s, those formed by dAGGGA and dTGGGT repeats display the lowest and highest *T*m values, respectively (52–58°C and 71°C; Figs 2 and 4). Yet, CD spectra reveal that dAGGGA G4s persist at high temperature, while dTGGGT G4s are completely unfolded at high temperature. This apparent discrepancy likely reflects differences in folding energetics (enthalpic and entropic contributions), molecularity and folding/unfolding kinetics.

In retrospect, the conformational landscape of dWGGGW repeats outlined in KCl provides a framework to better interpret the results of the NMM fluorescence assay. Specifically, lower NMM fluorescence intensities were observed with dTGGGTT and dAGGGA repeats compared to dTGGGT, dTGGGA, and dTGGGA repeats (Fig. 1B). NMM fluorescence enhancement upon binding to G4s is known to be conformation-dependent: parallel G4s yield the strongest enhancement (about 60-fold), antiparallel G4s the weakest (about 10-fold), and hybrid G4s an intermediate enhancement (about 40-fold) [54], reflecting NMM’s differential affinity for these G4 conformations [55]. This property accounts for the relatively low NMM fluorescence observed with the reference dTGGGTT repeats, which adopt a hybrid G4 conformation as indicated by their CD spectra (Supplementary Fig. S1). In contrast, dWGGGW repeats predominantly form parallel G4 structures (Fig. 2), resulting in a stronger NMM fluorescence emission. For dAGGGA repeats, the relatively low NMM fluorescence likely reflects the equilibrium between the G4 and the duplex structures in KCl solutions, with only a fraction of dAGGGA repeats (about 50% in KCl 100 mM) folded into G4s and available for NMM binding.

**Figure 9.**
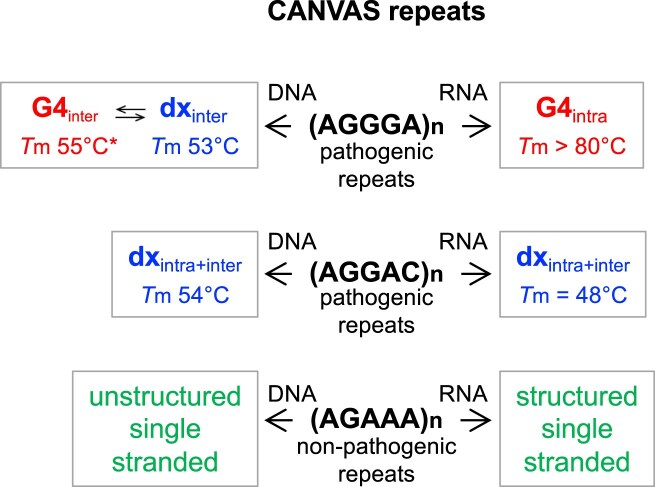
Conformational landscape of DNA and RNA CANVAS repeats. This conformational landscape refers to results obtained in 100 mM KCl. The *T*m values are those obtained for *n* = 12. The symbol “*” next to *T*m values indicates that fraction of G4s persists at 85°C. The intermolecular character of dAGGGA repeats duplexes (dx) suggests a parallel strand orientation, whereas the formation of both inter- and intramolecular structures by dAGGAC repeats suggests an antiparallel strand orientation.

The high degree of structural polymorphism exhibited by CANVAS pathogenic dAGGGA repeats in K^+^ is markedly reduced in RNA repeats and in the second pathogenic dAGGAC motif (Fig. 9). We confirmed that **rAGGGA** repeats fold into highly stable G4s (with *T*m values above 80°C) and we demonstrated that **d(AGGAC)_8,12,16_** and **r(AGGAC)_8,12_** repeats fold into both intra- and intermolecular duplexes with *T*m values above the human physiological temperature (above 49°C for DNA and 46°C for RNA). In contrast, the nonpathogenic **dAGAAA** repeats remain unstructured (in the absence of magnesium ions), while the respective **rAGAAA** repeats likely fold into a single-stranded helical conformation, based on the stacking of the purine bases, as proposed for poly(A) sequences [49]. In the presence of Mg^2+^, d(AGAAA)_12_, which closely mimics the 11-repeats configuration commonly found in healthy individuals, undergoes folding. Although we do not have sufficient data to propose a structural model, the melting profile, characterized by a sharp melting transition at 35°C, show that dAGAAA repeats are mostly unstructured at the human physiological temperature, even in the presence of Mg^2+^.

*In vitro*, the G4 structures formed by four and nine rAGGGA repeats have been shown to interact with RNA binding proteins [56]. Whether rAGGGA or rUCCCU RNA repeats are involved in CANVAS pathogenesis remains a matter of debate. Results on the detection of rAGGGA and rUCCCU repeats in cells from CANVAS patients are not conclusive [11, 20, 57]. Nevertheless, the detection of poly(KGREG) peptides (corresponding to translated rAGGGA repeats) in neurons from three CANVAS patients has been recently reported [20]. Interestingly, the behaviour of rAGGGA, rAGGAC and rAGAAA repeats, artificially overexpressed in a cell line, appeared to be very different: rAGGGA and rAGGAC formed foci, while rAGAAA did not form aggregates [26]. In light of these finding and of our results about the structures of CANVAS RNA repeats, it is tempting to speculate that the formation of foci might be related to the folding potential of rAGGGA and rAGGAC repeats, and driven by the interaction of their structures with RNA interaction proteins.

Returning to our original question—whether the folding behaviour of dAGGGA repeats diverges from that of the other dWGGGW repeats—our findings might explain why the dAGGGA motif, uniquely among the dWGGGW family (to our knowledge), is implicated in pathogenic expansions. Structurally, under physiological K^+^ conditions, dAGGGA repeats differ from the other dWGGGW repeats in at least two key ways: (i) in K^+^, dAGGGA repeats adopt both G4 and noncanonical duplex structures, while the other dWGGGW repeats fold exclusively into G4s; (ii) with respect to the G4 conformation, unlike the other dWGGGA repeats, dAGGGA repeats are not prone to fold into intramolecular G4s, folding exclusively into intermolecular G4s. These observations lead us to speculate that noncanonical duplexes, rather than G4s, may be the structures involved in the pathogenic expansion of dAGGGA repeats. This hypothesis is supported by the behaviour of the second pathogenic motif, dAGGAC, which shows a clear propensity to form duplexes but not G4s (Supplementary Fig. S12 illustrates the potential structures formed by dWGGGW and CANVAS repeats in a duplex context: dWGGGW beads-on-a-string-like parallel G4s, dAGGGA rod-like parallel G4s, dAGGGA parallel duplexes and dAGGAC hairpins). It is worth noting that parallel G4s and parallel duplexes represent only part of the structural polymorphism exhibited by dAGGGA repeats, as the formation of purine triplexes, where a third dAGGGA strand binds antiparallel to the AGGGA strand of the Watson–Crick duplex, has also been reported [30]. In a cellular context, CANVAS repeats may fold when transiently exposed as single-stranded DNA, such as during transcription and lagging-strand replication. During these cellular processes, the formation of stable duplexes and triplex structures appears plausible.

dAGGGA repeats represent an extreme case of a variant STR motif that gives rise to structural polymorphism, as demonstrated in this study, and genomic instability. In a previous study, we reported a milder case of such structural polymorphism, involving arrays of the unstable variant telomeric motif dGGGCTA embedded within canonical telomeric dGGGTTA repeats [43]. Unlike GGGTTA canonical repeats that fold exclusively in G4s, GGGCTA variant repeats adopted both G4 and hairpin conformations. While the G4s formed by both canonical GGGTTA repeats and variant GGGCTA repeats were efficiently unwound by the replication protein A (RPA), the GGGCTA hairpins were more resistant to RPA unwinding, suggesting that hairpin formation and reduced RPA binding may underlie the length-dependent instability of GGGCTA repeats observed in human telomeres. Overall, our findings support the idea that length dependent instability of DNA repeats may originate from structural polymorphism, and that non-G4 structures should not be overlooked: G4s, while often considered as the “usual suspects” in promoting genomic instability, are not necessarily the primary contributors of instability in all contexts.

The relationship between STR length dynamics and folding propensity is complex. An elegant study has provided insight into how the structural properties of STR repeats influence polymerase activity, and, in turn, contribute to the shaping of microsatellite length in eukaryotic genomes [4]. In summary, polymerase slippage at nonstructured STRs tends to promote repeat expansion and contraction, whereas polymerase stalling at structured STRs favours point mutations that disrupt the repeat pattern, thereby constraining further expansion. However, this model alone is not sufficient to explain the genetic data, which show that the nonpathogenic unstructured AGAAA repeats are more constrained in length than the pathogenic structured AGGGA repeats. As the authors note, polymerase slippage and error-prone synthesis alone cannot account for the STR expansions observed in neurological disorders, where more complex mechanisms must drive STR expansions beyond the length constraints imposed by polymerase slippage and error-prone replication [4].

## Conclusion

Our study uncovers the polymorphic structural landscape of dWGGGW repeats and provides a comprehensive understanding of the structures formed by CANVAS-associated DNA and RNA repeats. In particular, it highlights that the folding potential of pathogenic dAGGGA repeats is not limited to G4s but encompasses also homopurine duplex structures. Our research bridges genome instability and DNA/RNA structural polymorphism: by exploring the alternative folding modes of a repetitive purine motif, it sheds light on how sequence-encoded structural diversity might contribute to the function or dysfunction of repetitive elements in the genome. Once again, our findings illustrate the prophetic statement by Alex Rich: “DNA comes in many forms” [58].

## Supplementary Material

gkag051_Supplemental_File

## Data Availability

The authors confirm that the data supporting the findings of this study are available within the article and its supplementary data. Not shown data are available from the corresponding author P.A. on request.
