# Supplementary material for: Pentanucleotide guanine-rich WGGGW repeats, including CANVAS AGGGA repeats, form a variety of noncanonical structures
Source: Nucleic Acids Res. 2026 Jan 30;54(3):gkag051. doi: 10.1093/nar/gkag051 (PMC12856209; doi:10.1093/nar/gkag051)
Supplement: gkag051_Supplemental_File [file gkag051_supplemental_file.pdf]

Supplementary Data

Pentanucleotide guanine-rich WGGGW repeats, including CANVAS AGGGA repeats, form a variety of non-canonical structures

Jiawei Wang[1], Dehui Qiu[2], Jun Zhou[2], Jean-Louis Mergny[1*] & Patrizia Alberti[3*]

[1] Laboratoire d'Optique et Biosciences, Ecole Polytechnique, CNRS, INSERM, Institut Polytechnique de Paris, 91120 Palaiseau cedex, France.

[2] State Key Laboratory of Analytical Chemistry for Life Science, School of Chemistry and Chemical Engineering, Nanjing University, Nanjing 210023, P.R. China

[3] Laboratoire Structure et Instabilité des Génomes, Muséum national d'Histoire naturelle, CNRS, INSERM, Sorbonne Université, 75005 Paris, France

* Correspondance should be addressed to P. Alberti (patrizia.alberti@mnhn.fr). Correspondance may also be addressed to J.L. Mergny (jean-louis.mergny@inserm.fr)

Table S1. Oligonucleotide sequences

Name	Sequence (5'- 3')	Length (nt)	Chemistry
d(TGGGTT)$_4$	TGGGTTTGGGTTTGGGTTTGGGTT	20	DNA
d(TGGGTT)$_8$	TGGGTTTGGGTTTGGGTTTGGGTTTGGGTTTGGGTTTGGGTTTGGGTT	40	DNA
d(TGGGTT)$_{12}$	TGGGTTTGGGTTTGGGTTTGGGTTTGGGTTTGGGTTTGGGTTTGGGTTTGGGTTTGGGTTTGG<br>GTTTGGGTT	60	DNA
d(TGGGTT)$_{16}$	TGGGTTTGGGTTTGGGTTTGGGTTTGGGTTTGGGTTTGGGTTTGGGTTTGGGTTTGGGTTTGG<br>GTTTGGGTTTGGGTTTGGGTTTGGGTTTGGGTT	80	DNA
d(TGGGT)$_4$	TGGGTTGGGTTGGGTTGGGT	20	DNA
d(TGGGT)$_8$	TGGGTTGGGTTGGGTTGGGTTGGGTTGGGTTGGGTTGGGT	40	DNA
d(TGGGT)$_{12}$	TGGGTTGGGTTGGGTTGGGTTGGGTTGGGTTGGGTTGGGTTGGGTTGGGTTGGGTTGGGT	60	DNA
d(TGGGT)$_{16}$	TGGGTTGGGTTGGGTTGGGTTGGGTTGGGTTGGGTTGGGTTGGGTTGGGTTGGGTTGGGTTG<br>GGTTGGGTTGGGTTGGGT	80	DNA
d(TGGGA)$_4$	TGGGATGGGATGGGATGGGA	20	DNA
d(TGGGA)$_8$	TGGGATGGGATGGGATGGGATGGGATGGGATGGGATGGGA	40	DNA
d(TGGGA)$_{12}$	TGGGATGGGATGGGATGGGATGGGATGGGATGGGATGGGATGGGATGGGATGGGATGGGA	60	DNA
d(TGGGA)$_{16}$	TGGGATGGGATGGGATGGGATGGGATGGGATGGGATGGGATGGGATGGGATGGGATGGGATG<br>GGATGGGATGGGATGGGA	80	DNA
d(AGGGT)$_4$	AGGGTAGGGTAGGGTAGGGT	20	DNA
d(AGGGT)$_8$	AGGGTAGGGTAGGGTAGGGTAGGGTAGGGTAGGGTAGGGT	40	DNA
d(AGGGT)$_{12}$	AGGGTAGGGTAGGGTAGGGTAGGGTAGGGTAGGGTAGGGTAGGGTAGGGTAGGGTAGGGT	60	DNA
d(AGGGT)$_{16}$	AGGGTAGGGTAGGGTAGGGTAGGGTAGGGTAGGGTAGGGTAGGGTAGGGTAGGGTAGGGTAG<br>GGTAGGGTAGGGTAGGGT	80	DNA
d(AGGGA)$_4$	AGGGAAGGGAAGGGAAGGGA	20	DNA
d(AGGGA)$_8$	AGGGAAGGGAAGGGAAGGGAAGGGAAGGGAAGGGAAGGGA	40	DNA
d(AGGGA)$_{12}$	AGGGAAGGGAAGGGAAGGGAAGGGAAGGGAAGGGAAGGGAAGGGAAGGGAAGGGAAGG<br>GA	60	DNA
d(AGGGA)$_{16}$	AGGGAAGGGAAGGGAAGGGAAGGGAAGGGAAGGGAAGGGAAGGGAAGGGAAGGGAAGG<br>GAAGGGAAGGGAAGGGAAGGGA	80	DNA
d(AGAAA)$_4$	AGAAAAGAAAAGAAAAGAAA	20	DNA
d(AGAAA)$_8$	AGAAAAGAAAAGAAAAGAAAAGAAAAGAAAAGAAAAGAAA	40	DNA
d(AGAAA)$_{12}$	AGAAAAGAAAAGAAAAGAAAAGAAAAGAAAAGAAAAGAAAAGAAAAGAAAAGAAAAGAAA	60	DNA
d(AGAAA)$_{16}$	AGAAAAGAAAAGAAAAGAAAAGAAAAGAAAAGAAAAGAAAAGAAAAGAAAAGAAAAGAAAA<br>GAAAAGAAAAGAAAAGAAA	80	DNA
d(AGGAC)$_4$	AGGACAGGACAGGACAGGAC	20	DNA
d(AGGAC)$_8$	AGGACAGGACAGGACAGGACAGGACAGGACAGGACAGGAC	40	DNA
d(AGGAC)$_{12}$	AGGACAGGACAGGACAGGACAGGACAGGACAGGACAGGACAGGACAGGACAGGACAGGAC	60	DNA
d(AGGAC)$_{16}$	AGGACAGGACAGGACAGGACAGGACAGGACAGGACAGGACAGGACAGGACAGGACAGGAC<br>AGGACAGGACAGGACAGGAC	80	DNA
r(AGGGA)$_4$	AGGGAAGGGAAGGGAAGGGA	20	RNA
r(AGGGA)$_8$	AGGGAAGGGAAGGGAAGGGAAGGGAAGGGAAGGGAAGGGA	40	RNA
r(AGGGA)$_{12}$	AGGGAAGGGAAGGGAAGGGAAGGGAAGGGAAGGGAAGGGAAGGGAAGGGAAGGGAAGG<br>GA	60	RNA
r(AGAAA)$_4$	AGAAAAGAAAAGAAAAGAAA	20	RNA
r(AGAAA)$_8$	AGAAAAGAAAAGAAAAGAAAAGAAAAGAAAAGAAAAGAAA	40	RNA
r(AGAAA)$_{12}$	AGAAAAGAAAAGAAAAGAAAAGAAAAGAAAAGAAAAGAAAAGAAAAGAAAAGAAAAGAAA	60	RNA
r(AGGAC)$_4$	AGGACAGGACAGGACAGGAC	20	RNA
r(AGGAC)$_8$	AGGACAGGACAGGACAGGACAGGACAGGACAGGACAGGAC	40	RNA
r(AGGAC)$_{12}$	AGGACAGGACAGGACAGGACAGGACAGGACAGGACAGGACAGGACAGGACAGGACAGGAC	60	RNA
rTelHum21	GGGUUAGGGUUAGGGUUAGGG	21	RNA
F22	FAM-UGGCCCGUUCGCCCCUCCCGGG	22	RNA
37Q	GGGUUGCGGAGGGUGGGCCUGGGAGGGGUGGUGGCCA-BHQ1	37	RNA
VEGF	CGGGGCGGGCCTTGGGCGGGGT	22	DNA
ss20	CGTGTCGAAAGAGCTCCGAT	20	DNA
ss23	GTCGCCGGGCCAGTCGTCCATAC	23	DNA
ds26	CAATCGGATCGAATTCGATCCGATTG	26	DNA
ds 20bp	strand 1: CGTGTCGAAAGAGCTCCGAT<br>strand 2: ATCGGAGCTCTTTCGACACG	20<br>20	DNA
ds 40bp	strand1: AGCGCTGCACTACTGTTACGAGTCTGAAATCCTTCGCTCT<br>strand2: AGAGCGAAGGATTTCAGACTCGTAACAGTAGTGCAGCGCT	40<br>40	DNA

d(TGGGTT)n in KCl

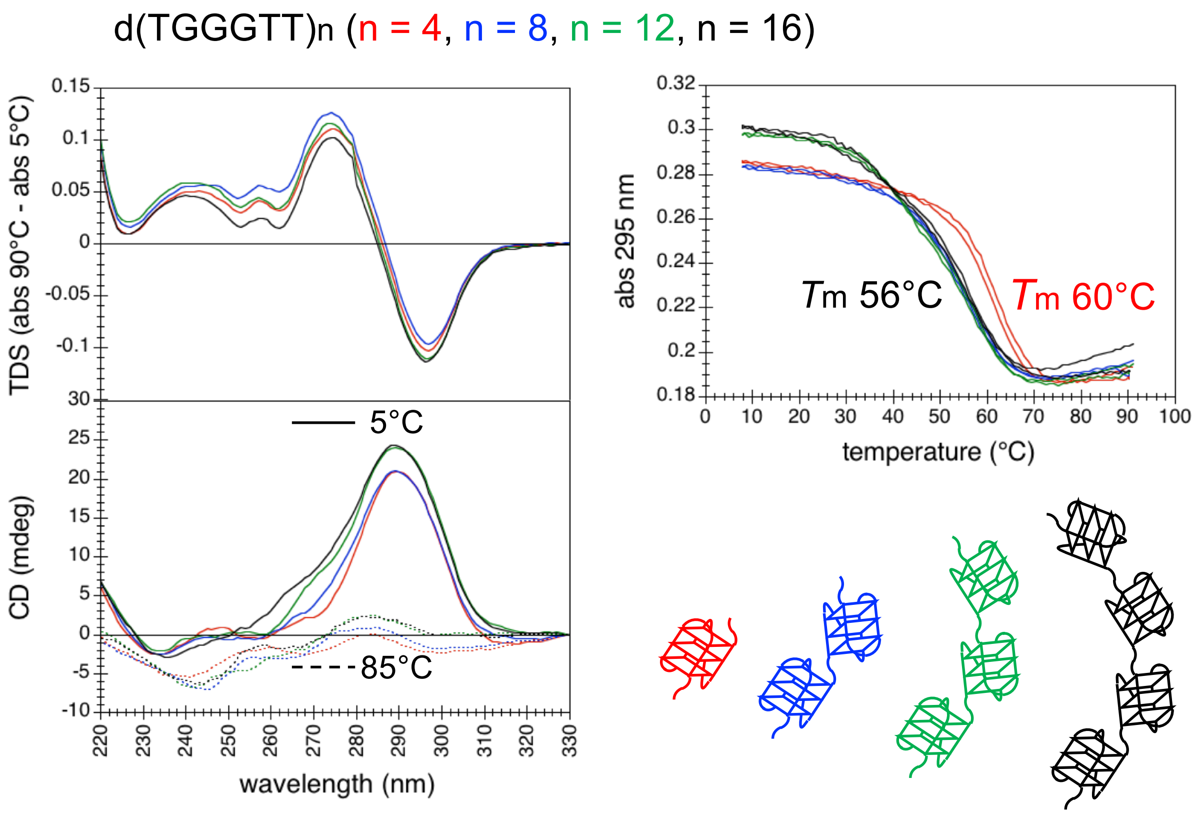

Figure S1
UV-spectroscopy investigation of dTGGGTT repeats

TDS, CD spectra at 5°C (solid lines) and 85°C (dotted lines) and absorbance at 295 nm (cooling and heating curves) as a function of temperature of d(TGGGTT)n (red: n = 4, blue: n = 8, green: n = 12, black: n = 16) in 100 mM KCl at strand concentrations of 6 μM for n = 4, 3 μM for n = 8, 2 μM for n = 12 and 1.5 μM for n = 16, corresponding to 6 μM of potential G4 units for each sequence.
Buffer: 10 mM cacodylic acid, pH 7.2 (LiOH).
Nearly identical TDS, CD spectra and melting curves, at strand concentrations corresponding to an identical concentration of potential G4 units, support the folding of d(TGGGTT)8,12,16 into 2, 3 and 4 similar and non-interacting contiguous G4 units, respectively, as we previously reported for GGGTTA repeats **(ref. 1)**. The shape of CD spectra support the folding of each G4 unit into a hybrid conformation.
The higher *T*m of the single G4 unit formed by d(TGGGTT)4 may be explained by stabilising interactions of the free T and TT nucleotides at the 5′ and 3′ end, respectively, with the G4 core. In a tandem context, these stabilizing interactions are likely impaired, since each internal TTT linker is shared by two adjacent G4 units **(ref. 1)**.
In 100 mM LiCl, TGGGTT repeats do not form stable structures (data not shown).

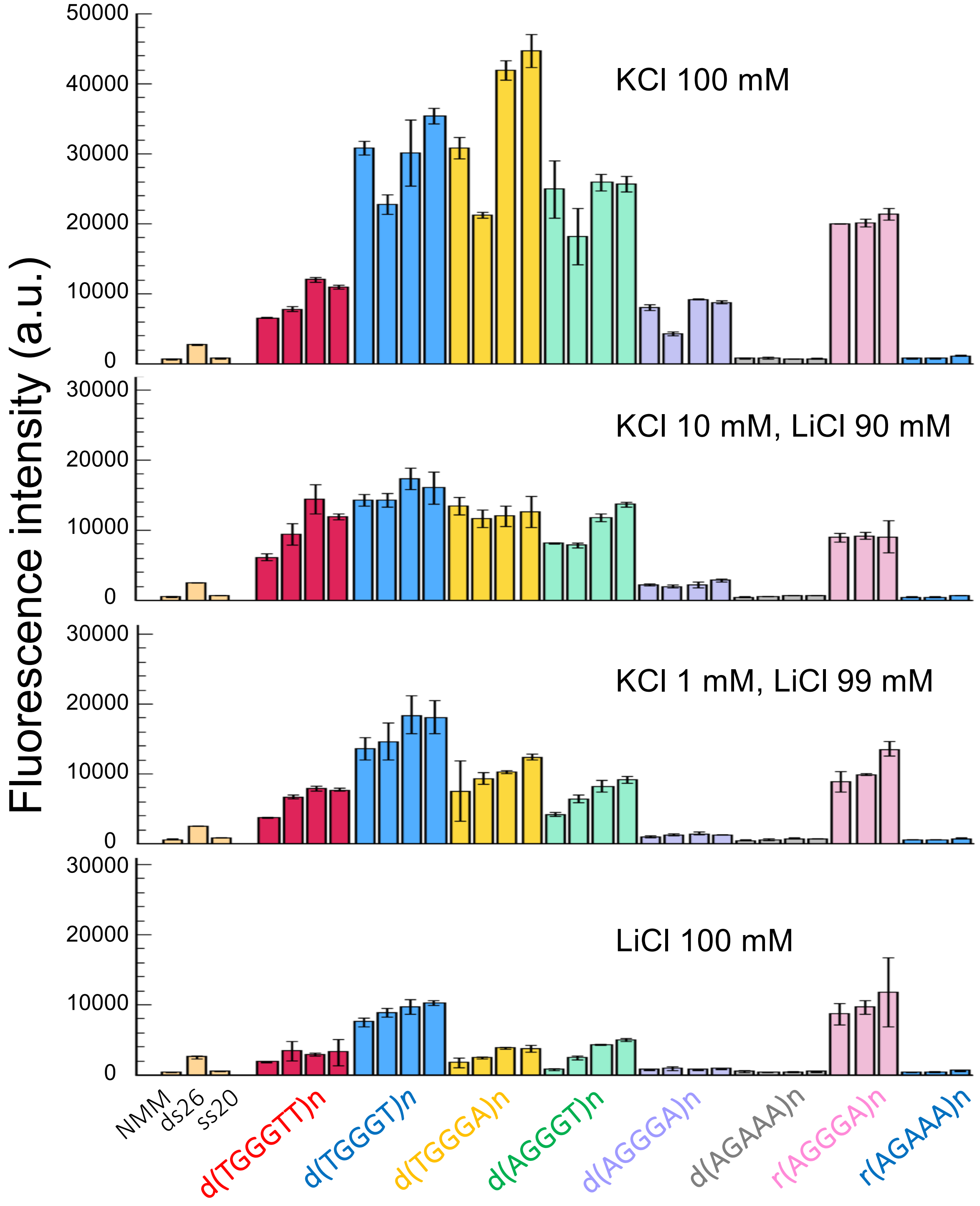

Figure S2

NMM fluorescence assay

Fluorescence emission of the G4 specific ligand NMM (5 µM) alone, in the presence of a double-stranded DNA (ds26, 2.5 µM), of a single-stranded DNA (ss20, 2.5 µM) and of pentanucleotide repeats (10/n µM , where n = 4, 8, 12, 16 for DNA oligonucleotides and n = 4, 8, 12 for RNA oligonucleotides), at KCl and LiCl concentrations reported in the figure. LiCl was added to keep the ionic strength constant while varying the KCl concentration.

Buffer: 10 mM cacodylic acid, pH 7.2 (LiOH).

An increase in NMM fluorescence indicates that at least a fraction of the oligonucleotide is folded into G4s.

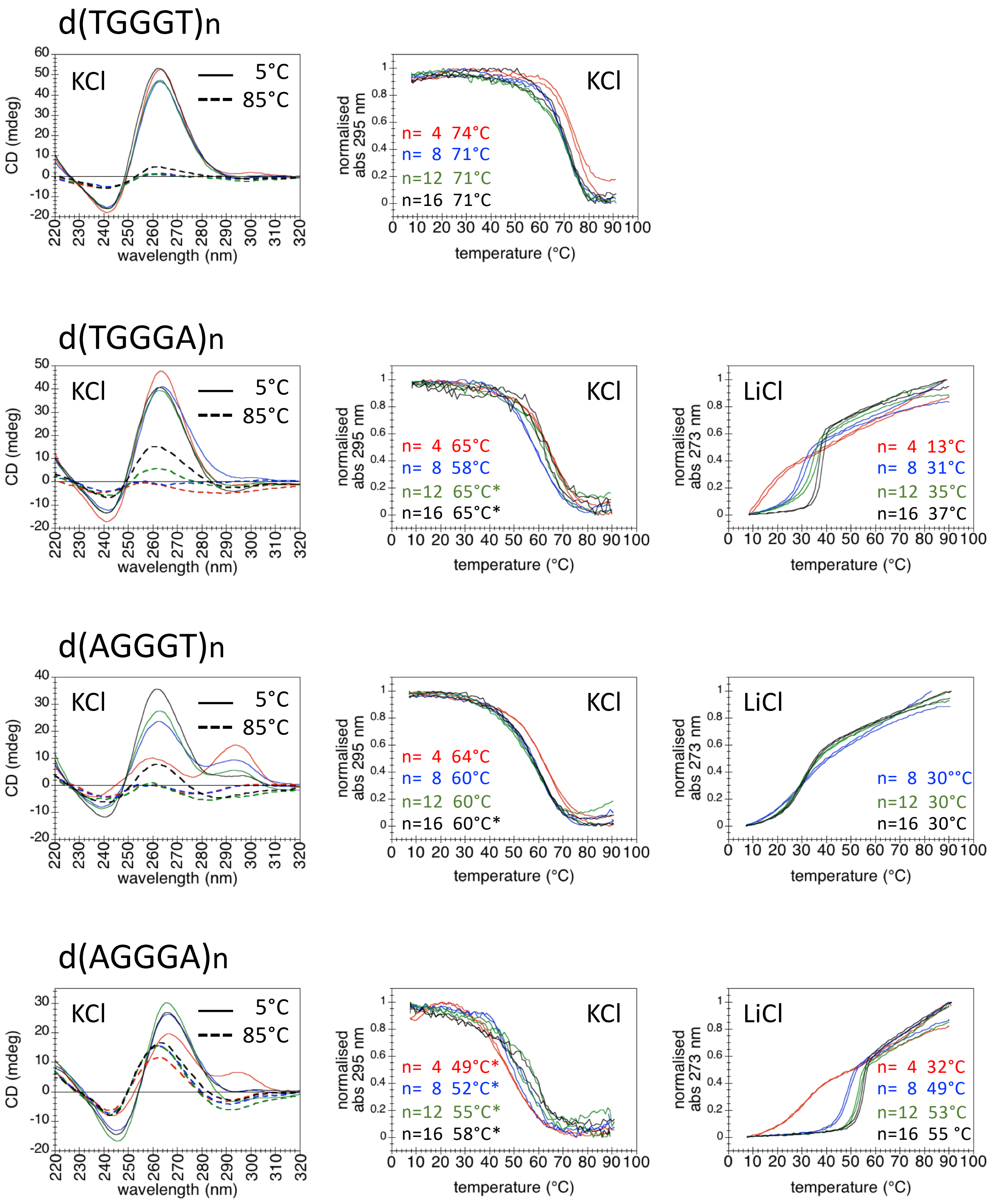

Figure S3

CD spectra and normalised melting curves of d(WGGGW)n repeats

CD spectra at 5°C (solid lines) and 95°C (dashed lines) in 100 mM KCl, and normalised absorbance as a function of temperature (cooling and heating curves) of d(WGGGW)n repeats (red: n = 4, blue: n = 8, green: n = 12, black: n = 16, strand concentration 24/n μM) in 100 mM KCl or LiCl. The symbol "*" next to T_m values indicates that the structure is not completely unfolded at 85°C, as assessed by CD spectra. T_m values of d(AGGGA)8,12,16 in KCl are midpoint value between $T_m^{coooling}$ and $T_m^{heating}$.

d(AGGGT)8,12,16 in KCl

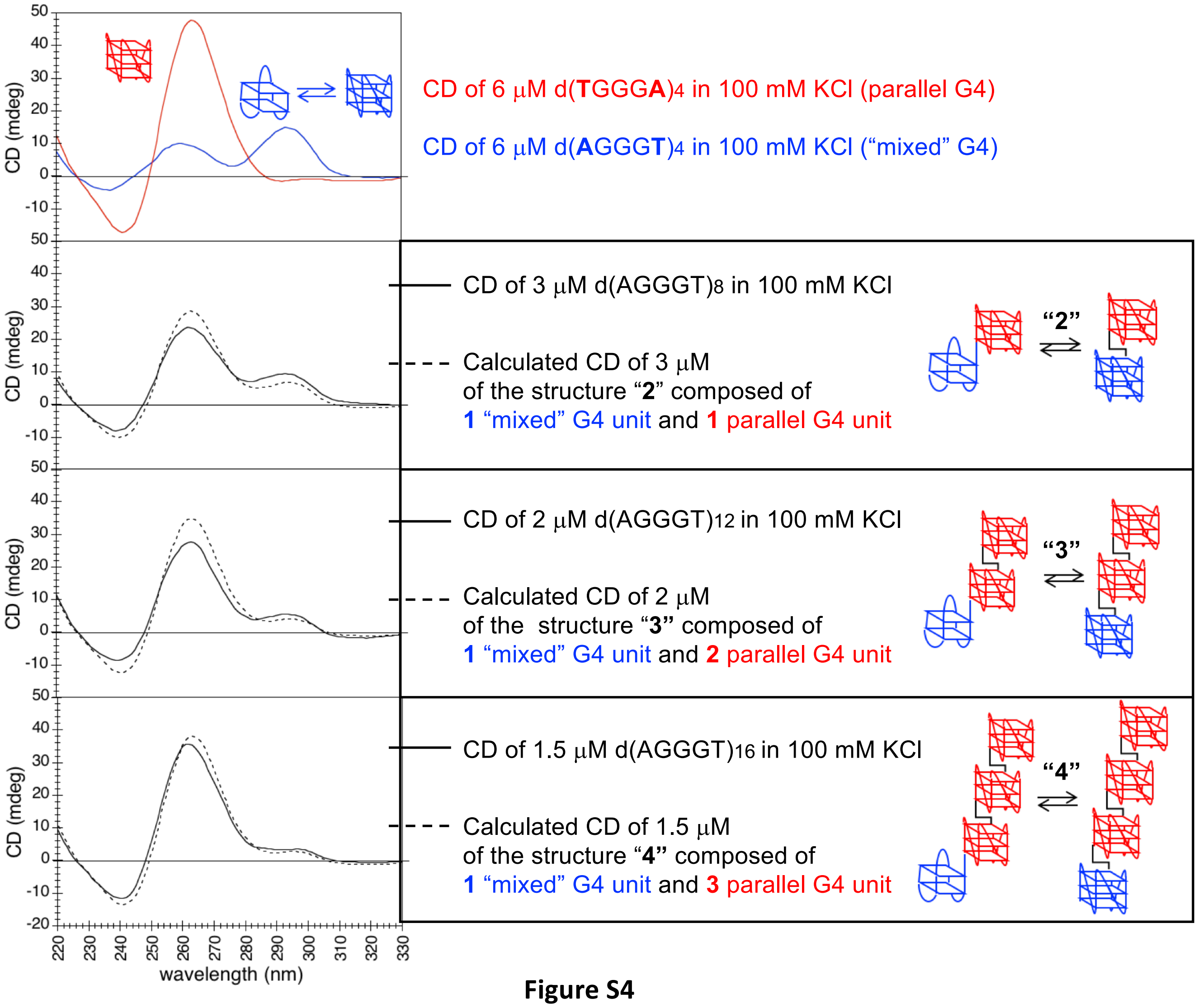

Figure S4

A possible structure of d(AGGGT)8,12,16 in KCl

The CD spectrum of d(AGGGA)4 in KCl is consistent with the formation of two distinct G4s reported in an article by Anh Tuân Phan’s team **(ref. 2)**: an antiparallel basket-type G4 with two G-tetrads and a parallel G4 with three G-tetrads. **Here, we refer to the coexistence of these two conformations as “mixed” conformation.**

Based on the evolution of CD spectra as the number of repeats increased to 8, 12 and 16 (an increase in the peak at 262 m and a decrease in the peak at 294 nm), we have formulated the hypothesis that d(AGGGT)8,12,16 could fold into one “mixed” G4 unit (blue unit) followed by 1, 2 and 3 parallel G4 units (red units), respectively, as shown in the figure. To test this hypothesis, we calculated the theoretical CD spectra of the structures “**2**”, “**3**” and “**4**” as the sum of the CD spectrum of the ”mixed” conformation (CD spectrum of d(AGGGT)4) and of the CD spectrum of a pure parallel conformation (CD spectrum of d(TGGGA)4) multiplied by 1, 2 and 3, respectively:

$$CD_{calculated}(3\mu M\ \text{"}\mathbf{2}\text{"}) = \frac{CD(6\mu M\ d(AGGGT)_4)}{6\mu M} * 3\mu M + \frac{CD(6\ \mu M\ d(TGGGA)_4)}{6\mu M} * 3\mu M * 1$$

$$CD_{calculated}(2\mu M\ \text{"}\mathbf{3}\text{"}) = \frac{CD(6\mu M\ d(AGGGT)_4)}{6\mu M} * 2\mu M + \frac{CD(6\mu M\ d(TGGGA)_4)}{6\ \mu M} * 2\mu M * 2$$

$$CD_{calculated}(1.5\mu M\ \text{"}\mathbf{4}\text{"}) = \frac{CD(6\mu M\ d(AGGGT)_4)}{6\mu M} * 1.5\mu M + \frac{CD(6\mu M\,d(TGGGA)_4)}{6\mu M} * 1.5\mu M * 3$$

The calculated spectra reproduce the measured spectra of d(AGGGT)8,12,16 quite satisfactorily, supporting our hypothesis.

d(AGGGT)4 in LiCl

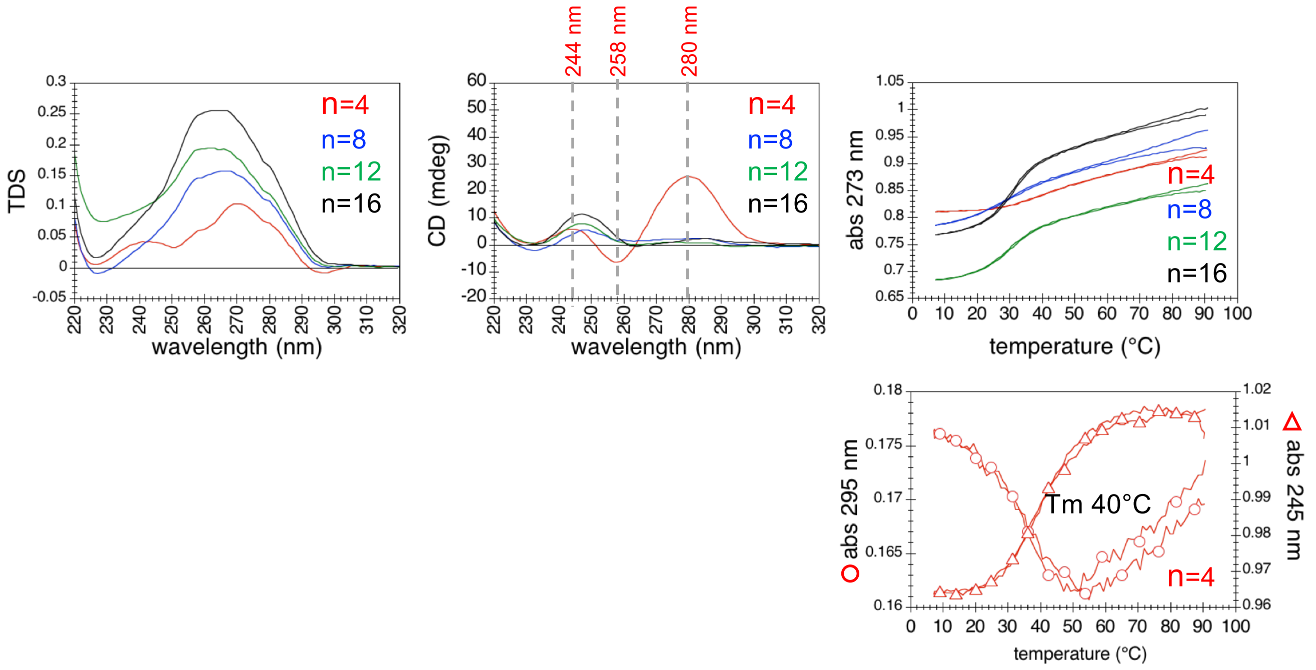

Figure S5
d(AGGGT)4 in LiCl

TDS, CD at 5°C and absorbance as a function of temperature (cooling and heating curves) of dAGGGT repeats in 100 mM LiCl. Buffer: 10 mM cacodylic acid, pH 7.2 (LiOH).

The TDS and the CD spectrum of d(AGGGT)4 are quite different from those of longer repeats. d(AGGGT)4 melting curves at 260 nm (not shown) and 273 nm do not have a clear transition, while at 295 nm and 245 nm they have a transition around 40°C. Although d(AGGGT)4 TDS is reminiscent of G4 structures, its CD spectrum does not exhibit the typical shapes of G4 CD spectra (*e.g.* antiparallel G4 have a minimum around 265 nm and a maximum around 295 nm).

d(AGGGGA)n	T_m heating (°C) at 295 nm	T_m cooling (°C) at 295 nm	T_m midpoint (°C)	hysteresis (°C)
4	46.7	44.4	45.6	2.3
5	47.8	44.2	46.0	3.6
8	53.4	44.2	48.8	9.2
12	61.1	47.5	54.3	13.6
16	63.9	48.0	56.0	15.9
20	65.2	48.2	56.7	17.0

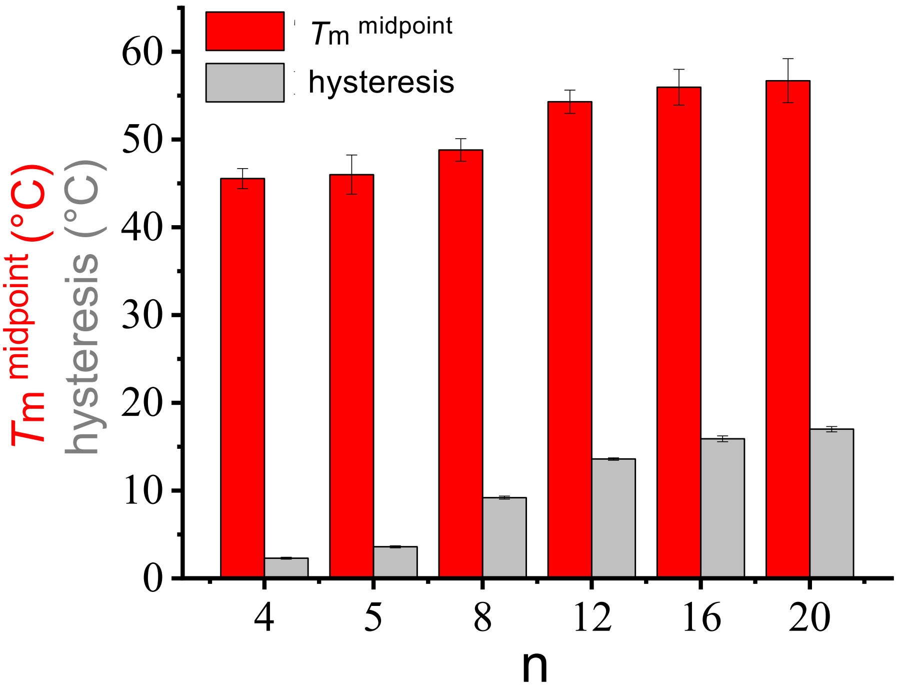

Figure S6
d(AGGGA)n melting temperatures and hysteresis

Midpoint melting temperatures, defined as $(T_m^{cooling} + T_m^{heating})/2$, of d(AGGGA)n repeats, determined at 295 nm, at 5 µM strand concentration, in a 10 mM Tris-HCl buffer pH 7.0 with 100 mM KCl, at a temperature variation rate of of 0.5°C min^{-1}.
Hysteresis is here defined as $T_m^{heating}$- $T_m^{heating}$.
$T_m^{midpoint}$ in this figure are 1°C to 3°C lower than those in Fig. 4A and hysteresis are more pronounced. This can be explained by the higher rate of temperature variation used for measurements in this figure (0.5°C min^{-1} versus 0.2°C min^{-1} for Fig. 4A).

d(AGGGA)12 in KCl

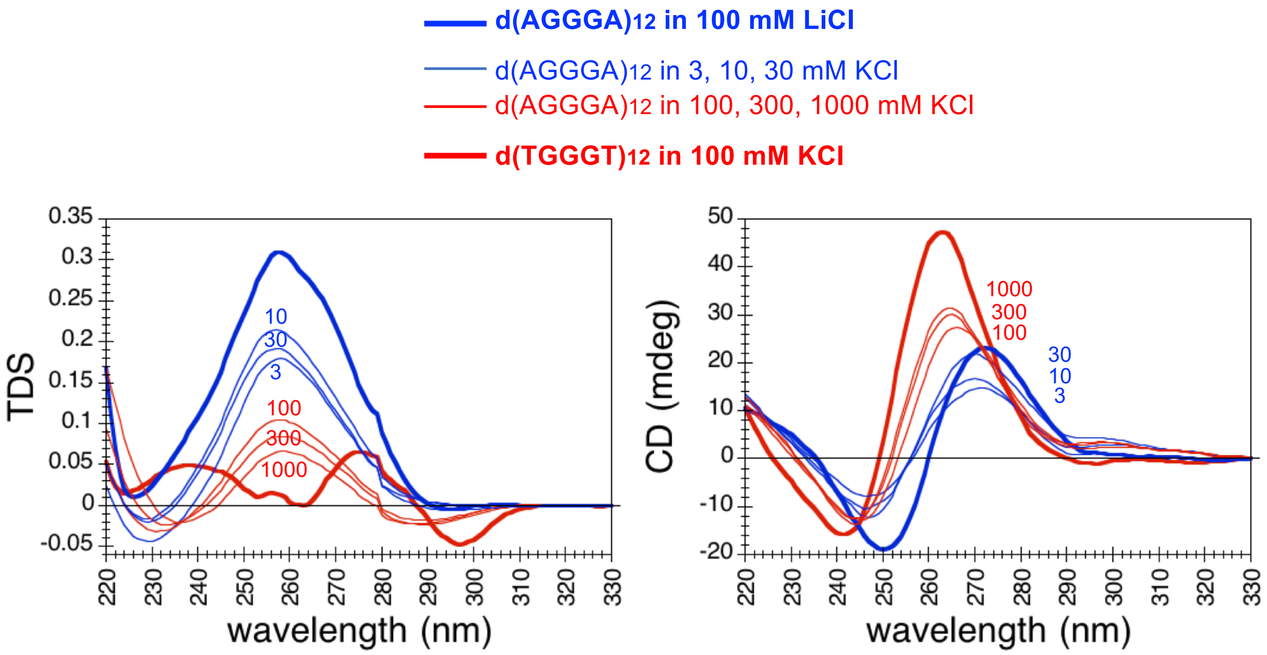

Figure S7

Effect of KCl concentration on the equilibrium between d(AGGGA)12 G4 and non-G4 structures

TDS and CD spectra of d(AGGGA)12 (3 μM) in 3, 10 and 30 mM KCl (thin blue lines) and in 100, 300 and 1000 mM KCl (thin red lines). Buffer: 10 mM cacodylic acid, pH 7.2 (LiOH).

The CD spectra of d(AGGGA)12 (3 μM) in 100 mM LiCl (thick blue line) and of d(TGGGT)12 (3 μM) in 100 mM KCl (thick red line) are the reference spectra of the non-G4 structure of d(AGGGA)12 and of a G4 parallel structure, respectively. CD spectra were recorded at 5°C, after annealing from 95°C to 5°C at 0.2°C min^{-1}.

At low KCl concentrations (3, 10 and 30 mM), the TDS and CD spectra of d(AGGGA)12 are shifted towards the non-G4 reference spectra, while at high KCl concentrations (100, 300 and 1000 mM), the TDS and CD spectra are shifted towards the G4 reference spectra.

d(AGGGA)16

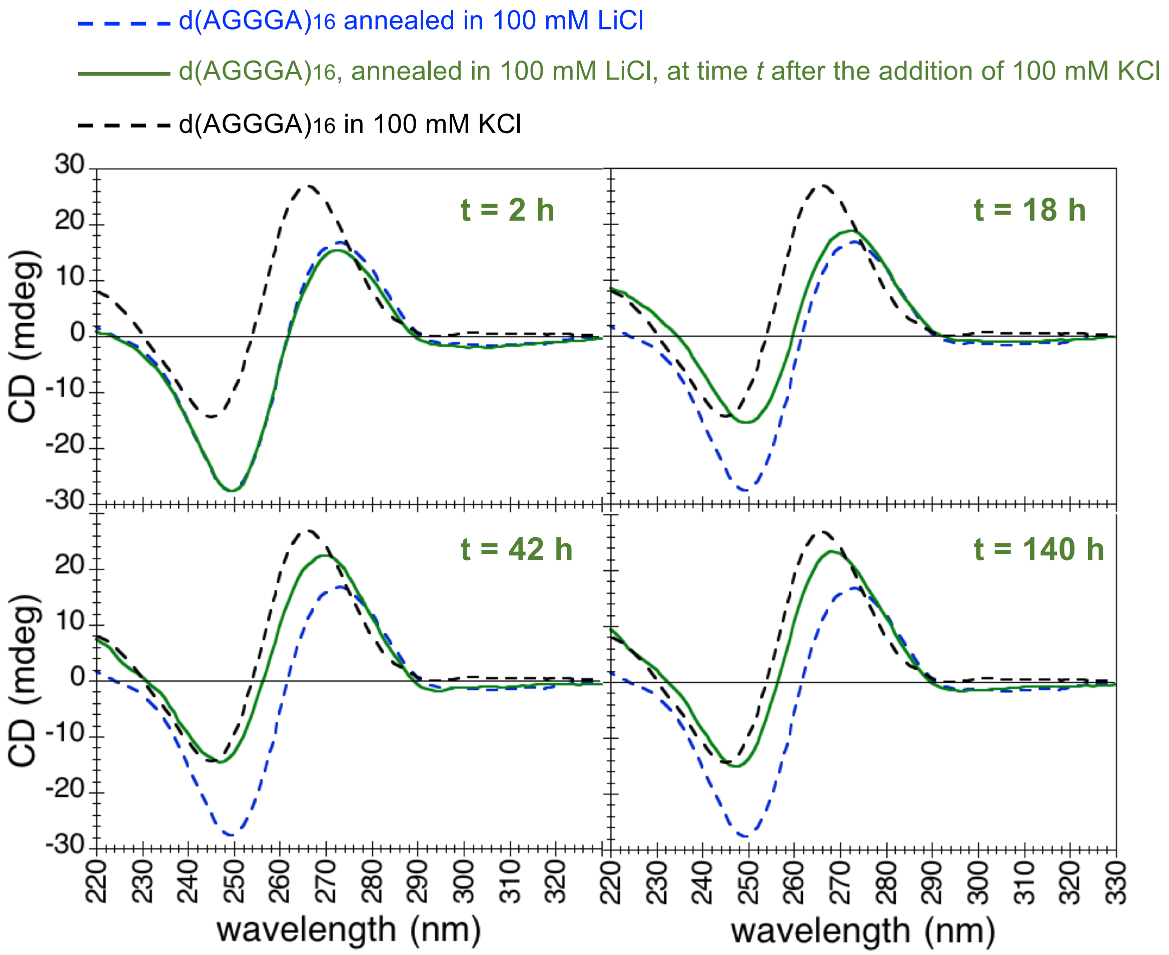

Figure S8

Conversion of the d(AGGGA)16 duplex structure toward the duplex/G4 equilibrium

Black dotted line: CD spectrum of d(AGGGA)16 (1.5 μM) annealed (from 95°C to 5°C at 0.4 °C min^{-1}) in a buffer containing 100 mM KCl (under this conditions the duplex and the G4 structures are in equilibrium, as discussed in the main text).

Blue dotted line: CD spectrum of d(AGGGA)16 (1.5 μM) annealed (from 95°C to 5°C at 0.4 °C min^{-1}) in a buffer containing 100 mM LiCl (under this conditions only the oligonucleotide is completely folded in the duplex structure, as discussed in the main text).

Green solid lines: CD spectra of the d(AGGGA)16 sample in 100 mM LiCl at time *t* after the addition of KCl at a final concentration of 100 mM (30 μL 2 M in 600 μL)

CD spectra were recorded at 5°C, the samples were stored at 5°C between two measurements. Buffer: 10 mM cacodylic acid, pH 7.2 (LiOH).

Similar results were obtained for d(AGGGA)8 and d(AGGGA)12 (data not shown).

d(AGGGA)8 in LiCl and in KCl

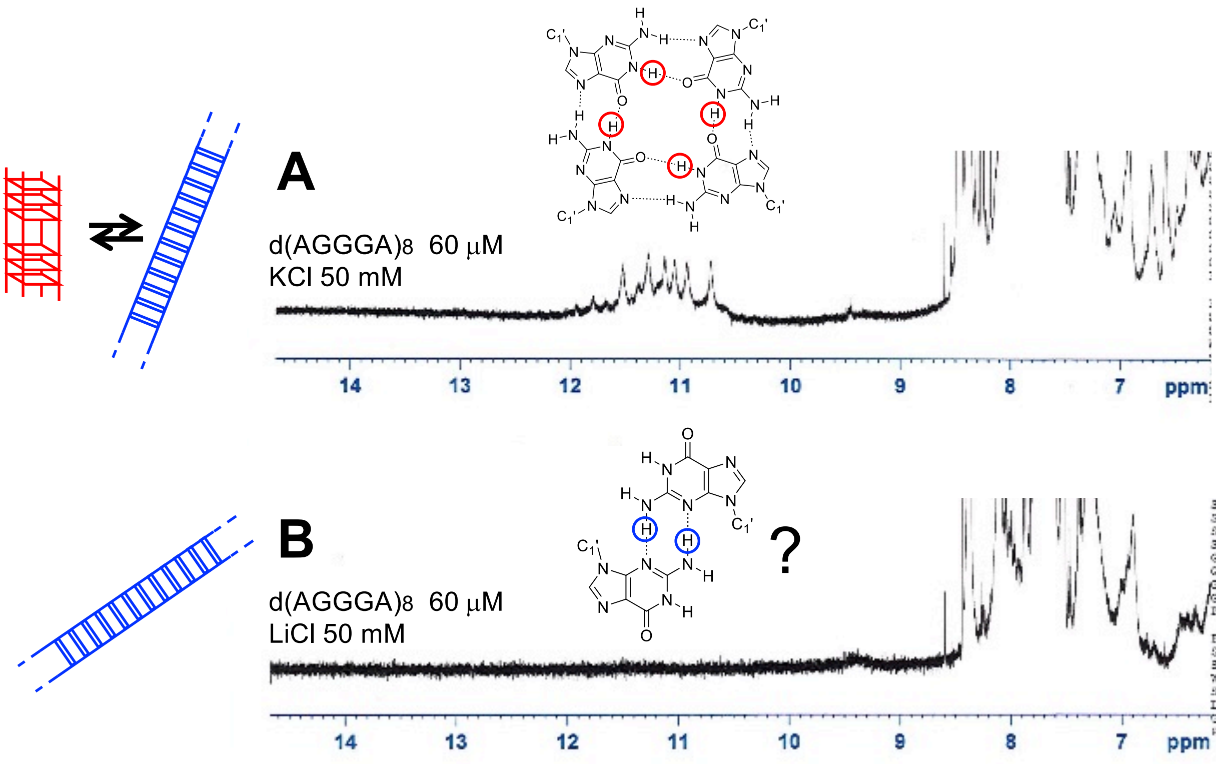

Figure S9
A possible G-G base-pairing in the duplex structure of dAGGGA repeats

NMR spectra of d(AGGGA)8 (60 µM) **(A)** in 50 mM KCl (where both the G4 and the duplex forms are present in equilibrium) and **(B)** in 50 mM LiCl (where only the duplex form is present), at 25°C. Buffer: 10 mM cacodylic acid, pH 7.2 (LiOH).

The NMR spectrum in LiCl exhibits no peak in the imino proton region. This suggests that the base pairs in dAGGGA duplexes might be based on hydrogen bonds involving only amino groups (such a possible base pairing between two guanines is shown in the figure). This also implies that the peaks in the imino proton region of the NMR spectrum in KCl are due to the G4 fraction only.

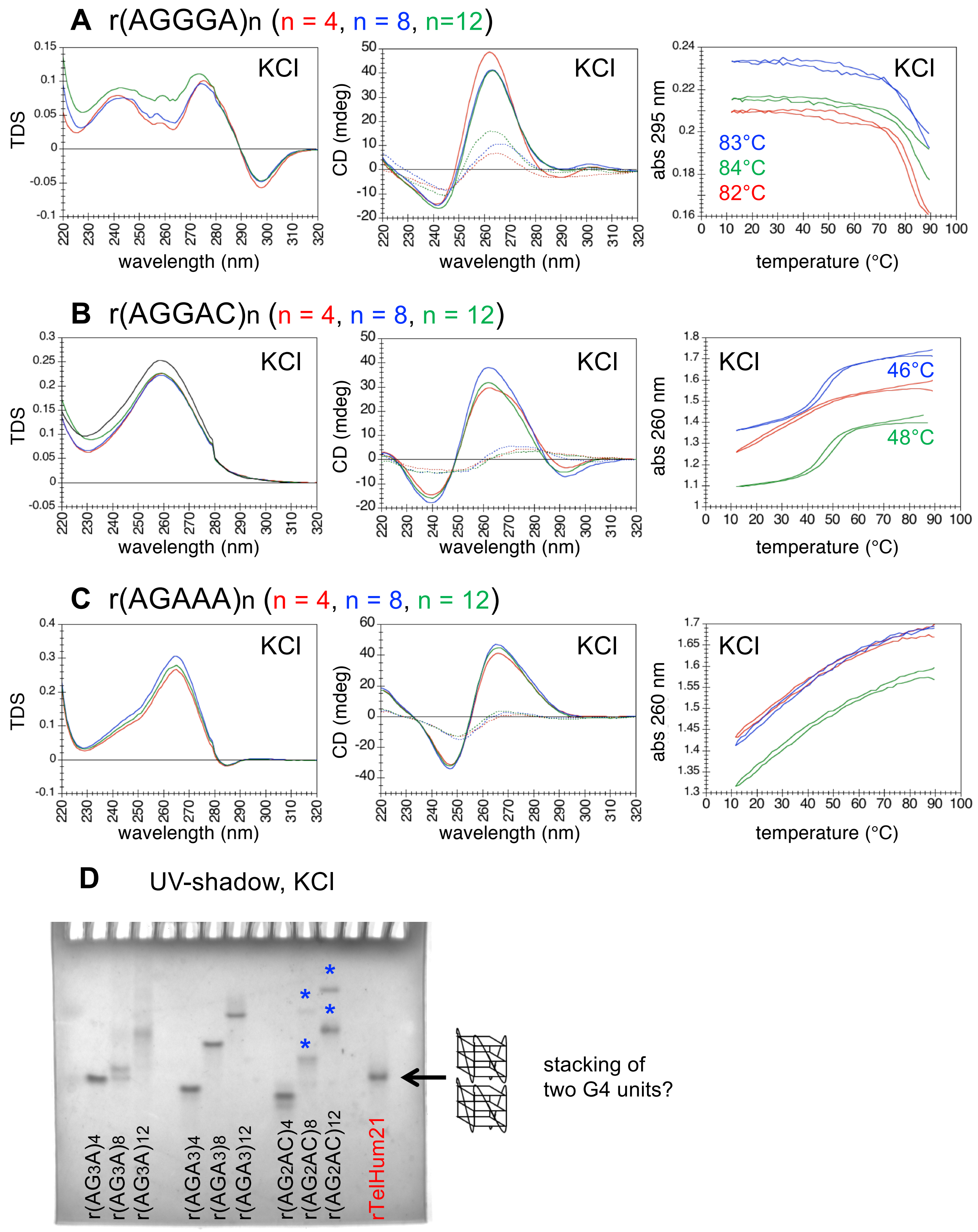

Figure S10

UV-spectroscopy and PAGE investigation of CANVAS RNA repeats in KCl

TDS, CD spectra (solid lines: 5°C, dotted lines 85°C) and absorbance as a function of temperature (cooling and heating curves) of r(AGGGA)n **(A)**, r(AGGAC)n **(B),** and d(AGAAA)n **(C)** (red: n = 4, blue: n = 8, green: n = 12, black: n = 16) in 100 mM KCl, at strand concentrations of 24/n μM (corresponding to 6 μM of potential G4 units or 18 μM of potential G-tetrads). *T*m values are reported. **(D)** Migration patterns of the RNA pentanucleotide repeats in 100 mM KCl, detected by UV-shadowing, at strand concentrations of 240/n μM . The symbol "*" marks the fast and the slow migrating bands detected for rAGGAC repeats. rTelHum21 is the oligonucleotide r(GGGUUA)3GGG. Electrophoresis was run in a cold room. Sample buffer: 10 mM cacodylic acid, pH 7.2 (LiOH).

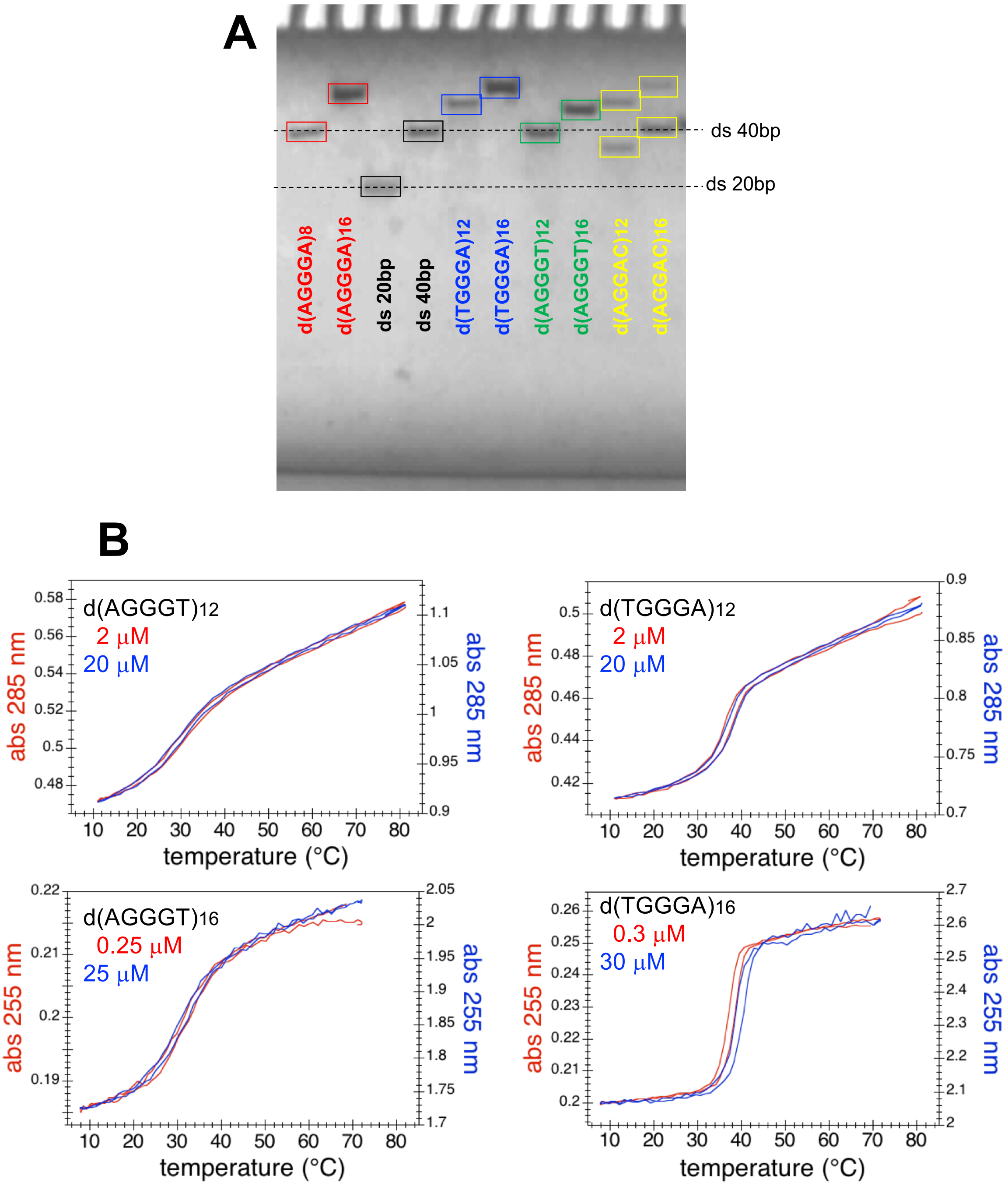

Figure S11

Investigation of the molecularity of DNA pentanucleotide repeats in LiCl

(A) PAGE migration pattern of DNA pentanucleotide repeats in 100 mM LiCl, detected by UV-shadowing, at strand concentrations of 240/n μM; ds 20bp and ds 40bp are intramolecular duplexes of 20 and 40 base pairs, respectively. The gel was run in a cold room.

(B) Absorbance as a function of temperature (cooling and heating curves) of dAGGGT and dTGGGA repeats in 100 mM LiCl, at strand concentrations spanning one and two orders of magnitude (upper and lower panels, respectively). Optical path length: 1 cm for 0.25, 0.3 and 2 μM samples (red curves), and 0.1 cm for 20, 25 and 30 μM samples (blue curves). Sample buffer: 10 mM cacodylic acid, pH 7.2 (LiOH). Temperature ramp rate: 0.4°C min^{-1}. Increasing the strand concentration by one order of magnitude did not affect the T_m of either AGGGT or TGGGA repeats. A two–order-of-magnitude increase led to a 2°C increase in the T_m of TGGGA repeats, while no change was observed for AGGGT repeats.

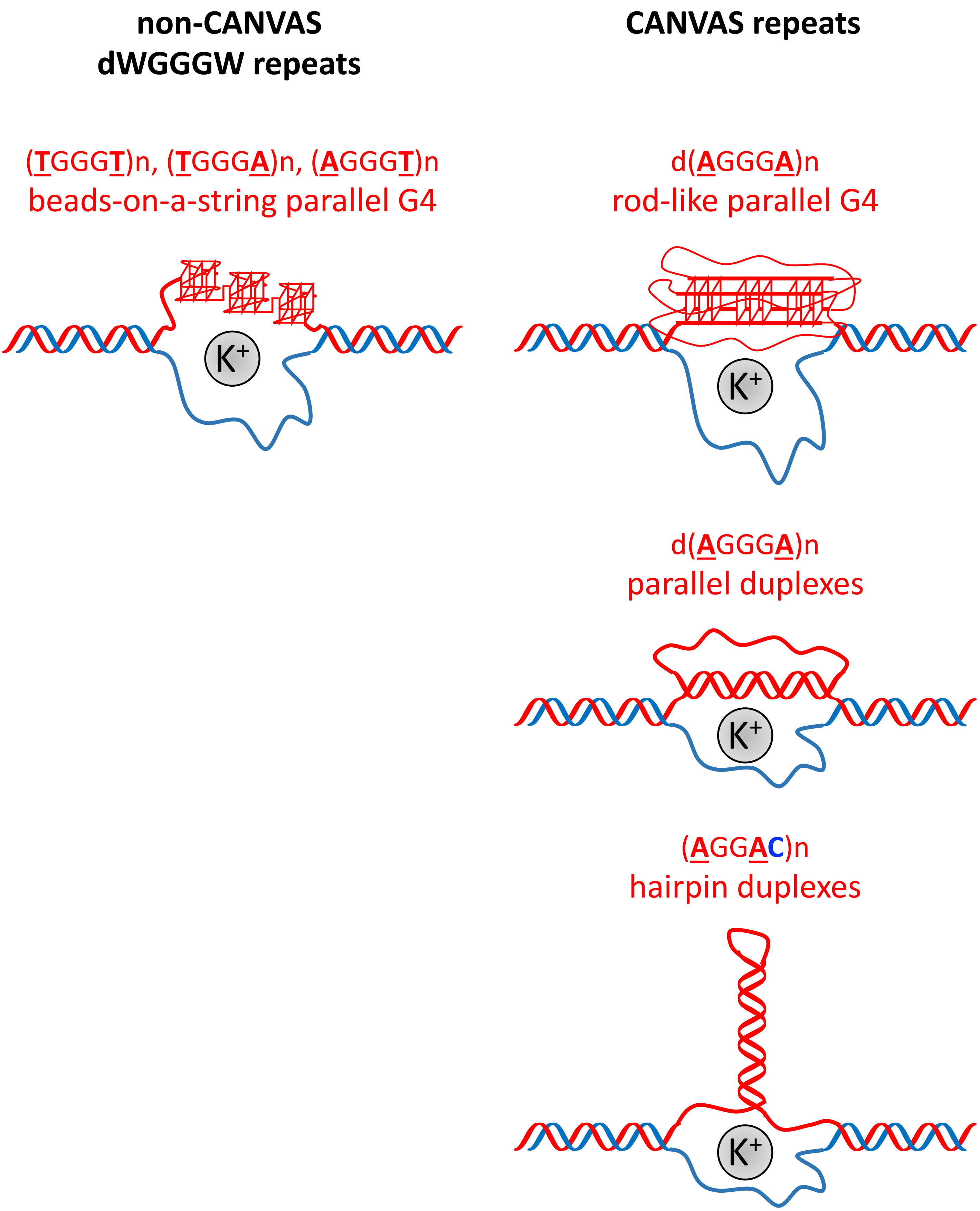

Figure S12

Potential structures formed by dWGGGW and CANVAS repeats in a duplex context

It is worth noting that while the stability of bead-on-a-string-like G4s is independent of the number of G4 units (an thus of the number of repeats), the stability of rod-like parallel G4, parallel duplexes and hairpins increases with the number of repeats.

REFERENCES

(1) Bugaut A, Alberti P.
Understanding the stability of DNA G-quadruplex units in long human telomeric strands.
Biochimie. 2015; 113:125-33. doi: 10.1016/j.biochi.2015.04.003.

(2) Hu L, Lim KW, Bouaziz S, Phan AT.
Giardia telomeric sequence d(TAGGG)4 forms two intramolecular G-quadruplexes in K+ solution: effect of loop length and sequence on the folding topology.
J Am Chem Soc. 2009;131(46):16824-31. doi: 10.1021/ja905611c.
